# Effect of Disulfiram and Copper Plus Chemotherapy vs Chemotherapy Alone on Survival in Patients With Recurrent Glioblastoma: A Randomized Clinical Trial

**DOI:** 10.1001/jamanetworkopen.2023.4149

**Published:** 2023-03-31

**Authors:** Katja Werlenius, Sara Kinhult, Tora Skeidsvoll Solheim, Henriette Magelssen, David Löfgren, Munila Mudaisi, Sofia Hylin, Jiri Bartek, Michael Strandéus, Magnus Lindskog, Havyan Bahroz Rashid, Louise Carstam, Sasha Gulati, Ole Solheim, Jiri Bartek, Øyvind Salvesen, Asgeir Store Jakola

**Affiliations:** 1 Department of Oncology, Sahlgrenska University Hospital, Gothenburg, Sweden; 2 Department of Oncology, Institute of Clinical Sciences, Sahlgrenska Academy, University of Gothenburg, Gothenburg, Sweden; 3 Department of Oncology, Department of Clinical Sciences, Lund University, Skåne University Hospital, Lund, Sweden; 4 Department of Clinical and Molecular Medicine, Faculty of Medicine and Health Sciences, Norwegian University of Science and Technology, Trondheim, Norway; 5 Cancer Clinic, St Olavs Hospital, Trondheim, Norway; 6 Department of Oncology, Oslo University Hospital, Oslo, Norway; 7 Department of Oncology, Faculty of Medicine and Health, Örebro University, Örebro, Sweden; 8 Department of Oncology, Linköping University Hospital, Linköping, Sweden; 9 The Finnmark Hospital, Hammerfest, Norway; 10 Department of Neurology, Karolinska University Hospital, Stockholm, Sweden; 11 Department of Neurosurgery, Karolinska University Hospital, Stockholm, Sweden; 12 Department of Clinical Neuroscience, Karolinska Institute, Stockholm, Sweden; 13 Department of Neurosurgery, Rigshospitalet, Copenhagen, Denmark; 14 Department of Oncology, County Hospital Ryhov, Jönköping, Sweden; 15 Department of Immunology, Genetics and Pathology, Uppsala University, Uppsala, Sweden; 16 Department of Pelvic Cancer, Section of Genitourinary Oncology, Karolinska University Hospital, Stockholm, Sweden; 17 Sahlgrenska Academy, University of Gothenburg, Gothenburg, Sweden; 18 Department of Neurosurgery, Sahlgrenska University Hospital, Gothenburg, Sweden; 19 Institute of Neuroscience and Physiology, Department of Clinical Neuroscience, Sahlgrenska Academy, University of Gothenburg, Gothenburg, Sweden; 20 Department of Neurosurgery, St Olavs Hospital, Trondheim, Norway; 21 Department of Neuromedicine and Movement Science, Norwegian University of Science and Technology, Trondheim, Norway; 22 Department of Medical Biochemistry and Biophysics, Division of Genome Biology, Science for Life Laboratory, Karolinska Institute, Stockholm, Sweden; 23 Danish Cancer Society Research Center, Copenhagen, Denmark; 24 Department of Public Health and Nursing, Norwegian University of Science and Technology, Trondheim, Norway

## Abstract

**Importance:**

Disulfiram has demonstrated broad antitumoral effect in several preclinical studies. One of the proposed indications is for the treatment of glioblastoma.

**Objective:**

To evaluate the efficacy and safety of disulfiram and copper as add-on to alkylating chemotherapy in patients with recurrent glioblastoma.

**Design, Setting, and Participants:**

This was a multicenter, open-label, randomized phase II/III clinical trial with parallel group design. Patients were recruited at 7 study sites in Sweden and 2 sites in Norway between January 2017 and November 2020. Eligible patients were 18 years or older, had a first recurrence of glioblastoma, and indication for treatment with alkylating chemotherapy. Patients were followed up until death or a maximum of 24 months. The date of final follow-up was January 15, 2021. Data analysis was performed from February to September 2022.

**Interventions:**

Patients were randomized 1:1 to receive either standard-of-care (SOC) alkylating chemotherapy alone, or SOC with the addition of disulfiram (400 mg daily) and copper (2.5 mg daily).

**Main Outcomes and Measures:**

The primary end point was survival at 6 months. Secondary end points included overall survival, progression-free survival, adverse events, and patient-reported quality of life.

**Results:**

Among the 88 patients randomized to either SOC (n = 45) or SOC plus disulfiram and copper (n = 43), 63 (72%) were male; the mean (SD) age was 55.4 (11.5) years. There was no significant difference between the study groups (SOC vs SOC plus disulfiram and copper) in 6 months survival (62% [26 of 42] vs 44% [19 of 43]; *P* = .10). Median overall survival was 8.2 months (95% CI, 5.4-10.2 months) with SOC and 5.5 months (95% CI, 3.9-9.3 months) with SOC plus disulfiram and copper, and median progression-free survival was 2.6 months (95% CI, 2.4-4.6 months) vs 2.3 months (95% CI, 1.7-2.6 months), respectively. More patients in the SOC plus disulfiram and copper group had adverse events grade 3 or higher (34% [14 of 41] vs 11% [5 of 44]; *P* = .02) and serious adverse events (41% [17 of 41] vs 16% [7 of 44]; *P* = .02), and 10 patients (24%) discontinued disulfiram treatment because of adverse effects.

**Conclusions and Relevance:**

This randomized clinical trial found that among patients with recurrent glioblastoma, the addition of disulfiram and copper to chemotherapy, compared with chemotherapy alone, resulted in significantly increased toxic effects, but no significant difference in survival. These findings suggest that disulfiram and copper is without benefit in patients with recurrent glioblastoma.

**Trial Registration:**

ClinicalTrials.gov Identifier: NCT02678975; EUDRACT Identifier: 2016-000167-16

## Key Points

**Question:**

Does the addition of disulfiram and copper to chemotherapy improve survival for patients with recurrent glioblastoma?

**Findings:**

In this randomized clinical trial of 88 patients with recurrent glioblastoma, the addition of disulfiram and copper to alkylating chemotherapy did not significantly improve survival at 6 months, as compared with alkylating chemotherapy only. Significantly more patients receiving disulfiram had adverse events of grade 3 or higher (34% vs 11%) and serious adverse events (41% vs 16%).

**Meaning:**

These findings suggest that the addition of disulfiram and copper to alkylating chemotherapy should not be recommended for patients with recurrent glioblastoma.

## Teaser

This randomized clinical trial investigates the efficacy and safety of disulfiram and copper as add-on to standard-of-care alkylating chemotherapy in patients with recurrent glioblastoma.

## Introduction

Repurposing of drugs to treat patients with cancer has emerged as a relevant approach.^1,2^ It is in this context that disulfiram, a drug used to treat alcohol dependency since 1947, has gained increased attention as a potential anticancer drug.^3,4,5^ In a wide range of preclinical studies disulfiram has demonstrated broad anticancer activity across tumor types.^3,6,7,8,9^

Glioblastoma (GB) is the most common and unfortunately also the most malignant of the diffuse gliomas.^10,11^ No major breakthrough in systemic treatment has occurred since the introduction of temozolomide.^12^ Several preclinical studies have suggested the benefit of disulfiram with or without copper supplementation against GB in vitro or in vivo.^4,8,13,14,15,16^ Suggested relevant mechanisms of action include: O^6^-methylguanine-DNA methyltransferase (MGMT) depletion,^15^ MGMT inhibition,^16^ radiosensitizing,^17,18^ inhibition of GB cancer stem cells,^13^ increased replication stress and DNA damage,^19^ antiangiogenic activity,^14^ and radical oxygen species leading to increased apoptotic activity.^4^ In addition, a study found that disulfiram plus copper caused dysfunction of NPL4, an essential cofactor of the p97/VCP segregase, which again severely impairs protein turnover and stress tolerance.^3^

The amount of clinical data on disulfiram with or without copper as an anticancer therapy does not yet match the preclinical evidence. A small phase II study in patients with lung cancer indicated limited adverse events with a dose of 120 mg disulfiram daily, and the study provided indications of prolonged survival.^20^ Another small study in cisplatin-responsive malignant neoplasms did not indicate any benefit of disulfiram.^21^ Of relevance in management of GB, a phase I study combining temozolomide with disulfiram in patients with GB indicated that up to 500 mg daily was tolerated.^22^

This randomized clinical trial was designed to further investigate the effect of disulfiram for recurrent GB. The study compared disulfiram and copper in combination with alkylating chemotherapy vs alkylating chemotherapy alone.

## Methods

### Trial Design and Oversight

This randomized clinical trial was an academic, open-label, 1:1 controlled phase II/III trial with parallel group design. Adult patients with first recurrence of GB were eligible for inclusion. The open-label study design was chosen as a pragmatic solution, as complete temperance was deemed too intrusive for the control group. Patients were recruited at 7 study sites in Sweden (Göteborg, Lund, Örebro, Linköping, Stockholm, Jönköping, and Uppsala) and 2 sites in Norway (Trondheim and Oslo), from January 16, 2017, until November 15, 2020.

The trial (NCT02678975) was conducted in accordance with International Council for Harmonization Good Clinical Practice guidelines. The study protocol and all amendments were approved by the Ethics Committee in Gothenburg (Regional Ethics Review Board) and by the Ethics Committee in region Central Norway. The study was also approved by the Swedish Medical Products Agency and by the Norwegian Medicines Agency. All included patients signed written informed consent prior to any study specific procedure. In Norway, monitoring was performed by the Clinical Trial Unit at Norwegian University of Science and Technology while the Clinical Trial Unit at Department of Oncology, Sahlgrenska University Hospital monitored the Swedish sites. An interim analysis by an independent Data Safety Monitoring Board was preplanned at 50% patient inclusion. This randomized clinical trial follows the Consolidated Standards of Reporting Trials (CONSORT) reporting guideline.

### Patients

Eligible patients were 18 years or older, had a previous, histologically verified diagnosis of GB, and presented with a first recurrence documented by magnetic resonance imaging (MRI). Key inclusion criteria were indication for alkylating chemotherapy, Karnofsky performance status score greater than or equal to 60, and willingness to refrain from alcoholic beverages if randomized to the experimental treatment with disulfiram. Radiotherapy within 3 months before diagnosis of progression was a main exclusion criteria to reduce the risk of including patients with so-called pseudoprogression after radiotherapy. Prior chemotherapy for progression or other experimental therapies for GB were not allowed. Additional inclusion and exclusion criteria are listed in Supplement 1 and published study protocol.^23^

### Randomization

The randomization was computer generated in a 1:1 ratio, with stratification for study center. As described earlier, randomization was web-based, using the system WebCRF 3.0 with blocks of varying sizes to make prediction of allocation impossible.^23^

### Interventions

Patients were randomly assigned, in a 1:1 ratio, to receive either any alkylating chemotherapy (temozolomide, lomustine, or the so-called PCV regimen that is a combination of procarbazine, lomustine, and vincristine) according to standard of care (SOC), or SOC together with disulfiram and nutritional copper supplement (SOC plus disulfiram and copper). Chemotherapy was given according to established treatment protocols. For patients randomized to SOC plus disulfiram and copper the administration of disulfiram and nutritional copper supplement started concomitant with the alkylating chemotherapeutic treatment. In the phase I study by Huang et al^22^ the maximum tolerated dose of disulfiram was determined to be 500 mg per day. In our study patients were to take disulfiram once daily, in the evening, as an oral dose of 400 mg. In case of intolerance, dose reduction to 200 mg per day was allowed. Copper supplement was administered once daily, separately from disulfiram, at a dose corresponding to 2.5 mg of elementary copper. Third line treatment after disease progression was allowed at the discretion of the investigator. Disulfiram and copper was to continue also after change of chemotherapy and following chemotherapy withdrawal due to reached cumulative dose or side effects. Crossover to treatment with disulfiram and copper was not allowed for patients randomized to SOC.

### Patient Evaluation and Follow-up

To reduce patient burden, timing of data collection was scheduled according to the choice of chemotherapy regimen, where patients who received temozolomide were assessed every 4 weeks, while patients treated with lomustine or PCV where assessed every 6 weeks. All patients in the study were assigned to undergo MRI and clinical examination at 3-month intervals as part of study protocol. Compliance of disulfiram and copper was assessed by tablet count at the study visits, in addition to patient self-reporting. Patient-reported health-related quality of life (HRQoL) was measured with EuroQol-5D-3L (EQ-5D),^24^ and assessed until tumor progression, or as long as on disulfiram and copper treatment, in case of treatment beyond progression. Patients were followed in the trial until death, for a maximum of 24 months, or until end of study (January 15, 2021). However, after withdrawal of antitumoral treatment all study-specific follow-up was terminated, in order to minimize patient burden in the end-of-life setting.

### Outcomes

The primary end point was survival at 6 months from the date of randomization. Secondary end points were overall survival from randomization, progression-free survival (PFS) and progression at 6 and 12 months. PFS was measured as the time from randomization to the date of investigator-assessed progressive disease (PD) according to the Response Assessment in Neuro-Oncology criteria,^25^ or death, whichever occurred first. Additional secondary end points were change in HRQoL, volumetric growth rate assessed from baseline MRI to first follow-up MRI scan,^26^ and safety assessed according to the Common Terminology Criteria for Adverse Events (CTCAE) version 4.0.^27^ Only grade 3, 4, and 5 toxic effects or grade 2 or greater infections were reported.

### Statistical Analysis

The sample size calculation assumed that the experimental group would have an improvement in the proportion achieving 6-month survival from 60% to 80%, with a final sample size of 128 patients needed (64 in each group; α = .10, power = 80%, and 2-sided test). We expected 10% attrition, thus the planned randomization was therefore 142, with 71 patients in each treatment group. The choices underlining these decisions are described in the protocol,^23^ and in the statistical analysis plan (Supplement 1).

All analyses were performed on the intention-to-treat population, unless otherwise specified. Comparison of proportions were performed with χ^2^ test. In time to event analyses, Kaplan-Meier plots were used for visualization and analyzed with Cox proportional hazard method. For HRQoL, an area under the curve approach was planned, but due to the high dropout from baseline to subsequent assessment we decided to analyze the change in EQ-5D index value from baseline to 3 and 6 months between groups using independent samples *t* test.^28^ Independent samples *t* test was also used to analyze between group differences in tumor volume, where percentage of daily change was estimated from baseline and first follow-up MRI. Given the potential for type 1 error due to multiple comparisons, findings for analyses of secondary end points should be interpreted as exploratory. Statistical analysis was performed February to September 2022 using R software version 4.2.2 with RStudio (2022.12.0+353) (R Project for Statistical Computing).

## Results

### Interim Analysis

The report of the interim analysis was received October 22, 2020, and included 84 patients, that is 13 patients more than the original planned analysis at 50% inclusion. The discrepancy was attributed to COVID-19. The interim analysis resulted in early termination of the study, as significantly more serious adverse events (SAEs) were reported in the experimental treatment group and there was low conditional power for any treatment benefit. The results presented here are from the final analysis of all randomized patients.

### Patient Characteristics

**Figure 1.**
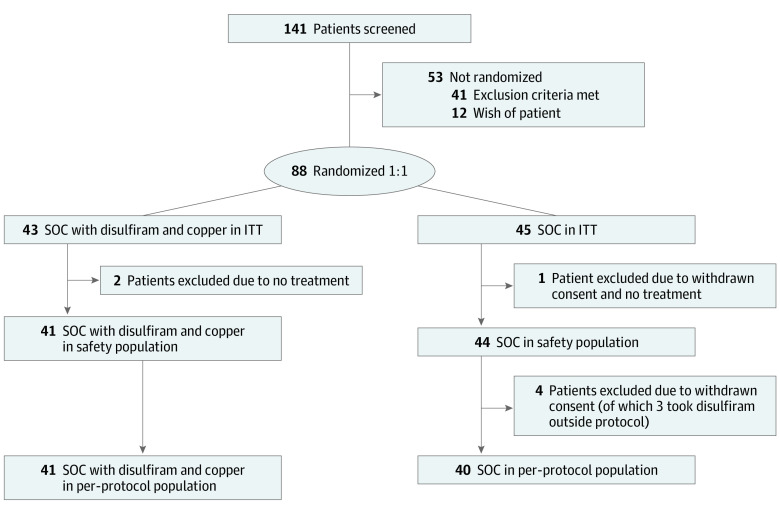
Randomization and Study Population ITT, intention-to-treat population; SOC, standard-of-care alkylating chemotherapy.

There were 141 patients screened and 88 patients randomized in the trial: 45 in the SOC group and 43 in the SOC plus disulfiram and copper group. Among the 88 patients randomized, the mean (SD) age was 55.4 (11.5) years; 63 (72%) were male, and 54 (61%) had Karnofsky performance status 90% to 100% at baseline. Three patients did not start any treatment within the trial and were excluded from the safety analysis. A total of 5 patients in the SOC group withdrew consent and were, together with patients not receiving any treatment, excluded from the per protocol (PP) population. The randomization and study populations are presented in Figure 1.

There were no statistically significant differences in baseline characteristics between the 2 treatment groups (Table 1). Corticosteroids use at baseline was similar in both treatment groups (51% vs 47%), as was the proportion of patients with known hypermethylation of the MGMT-promoter (29% vs 26%). A mutation in isocitrate dehydrogenase 1 (*IDH1*) gene was present in 7% of patients randomized to SOC and in 9% of patient randomized to SOC plus disulfiram and copper. Only one patient did not receive temozolomide treatment as part of the initial treatment and most patients (n = 82, 93%) underwent treatment with radiotherapy and concurrent temozolomide after the initial surgery (Table 1).

**Table 1.** Baseline Characteristics in the Intention-to-Treat Population

Characteristic	No. (%)^a^		
	SOC (n = 45)	SOC plus disulfiram and copper (n = 43)	Total (N = 88)
Age, mean (SD), years	54.7 (11.4)	56.2 (11.8)	55.4 (11.5)
Sex			
Male	30 (67)	33 (77)	63 (72)
Female	15 (33)	10 (23)	25 (28)
Karnofsky performance status			
60%	4 (9)	2 (5)	6 (7)
70%-80%	13 (29)	15 (35)	28 (32)
90%-100%	28 (62)	26 (60)	54 (61)
Initial surgery			
Resection	39 (87)	40 (93)	79 (90)
Biopsy	6 (13)	3 (7)	9 (10)
Initial radiotherapy with concurrent temozolomide			
Yes	41 (91)	41 (95)	82 (93)
No^b^	4 (9)	2 (5)	6 (7)
Tumor characteristics^c^			
IDH1			
Wildtype	33 (73)	30 (70)	63 (72)
Mutated	3 (7)	4 (9)	7 (8)
Unknown	9 (20)	9 (21)	18 (20)
MGMT			
Unmethylated	19 (42)	21 (49)	40 (46)
Methylated	13 (29)	11 (26)	24 (27)
Unknown	13 (29)	11 (26)	24 (27)
Use of steroids at baseline			
Yes	23 (51)	20 (47)	43 (49)
No	22 (49)	22 (51)	44 (50)
Missing		1 (2)	1 (1)

Abbreviations: IDH1, isocitrate dehydrogenase; MGMT, O^6^-methylguanine-DNA methyltransferase; SOC, standard-of-care alkylating chemotherapy.

^a^ Percentages may not add up to 100 due to rounding.

^b^ Other initial treatment than concurrent radiochemotherapy, only one patient did not receive temozolomide as part of the initial treatment.

^c^ At initial diagnosis.

### Treatment Characteristics

Except for the intervention, there were no statistically significant differences between the 2 study groups in treatment provided at time of recurrence (Table 2). In short, lomustine was the main alkylating agent used in both groups. In the SOC group, tumor resection for recurrence was performed in 24% (n = 11) compared with 37% (n = 16) in the SOC plus disulfiram and copper group (*P* = .19). Similar results were observed in the PP population (eTable 2 in Supplement 2). Reirradiation was provided to 5% of all patients. The median (IQR) duration of chemotherapy was significantly longer in the SOC group (93.5 days [58-210 days]) as compared with the SOC plus disulfiram and copper group (60 days [33-98]) (*P* = .007).

**Table 2.** Treatment Characteristics at First Recurrence in the Intention-to-Treat Population

Treatment characteristic	No. (%)		*P* value
	SOC (n = 45)	SOC plus disulfiram and copper (n = 43)	
Chemotherapy			
Temozolomide	12 (27)	14 (32)	.53
Lomustine	29 (64)	22 (51)	
PCV	3 (7)	5 (12)	
Not started	1 (2)	2 (5)	
Duration of chemotherapy, d			
No.	42	35	.007
Median (IQR)	93.5 (58-210)	60 (33-98)	
Other treatments for recurrence			
Radiotherapy	1 (2)	3 (7)	.28
Surgery	11 (24)	16 (37)	.19

Abbreviations: PCV, procarbazine, lomustine, and vincristine; SOC, standard-of-care alkylating chemotherapy.

### Survival

**Figure 2.**
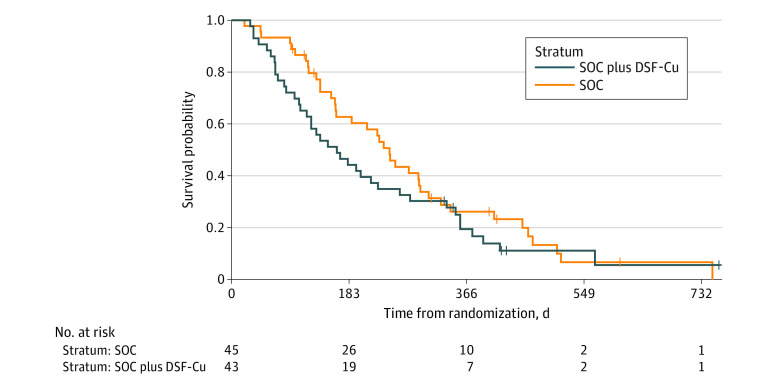
Overall Survival Time (From Randomization to Death) The difference between survival was not significant, *P* = .26, using a Cox proportional hazard method. DSF-Cu indicates disulfiram and copper; SOC, standard-of-care alkylating chemotherapy.

Three patients in the SOC group had shorter follow-up than the required 6 months for the primary end point, due to the early termination of the study. There was no significant difference in the primary outcome, namely survival at 6 months after randomization, with 62% (26 of 42 patients) alive in the SOC group compared with 44% (19 of 43 patients) in the group treated with SOC plus disulfiram and copper (*P* = .10). At 9, 12, and 24 months, the proportions alive were 46% (n = 18) vs 33% (n = 14) (*P* = .21), 27% (n = 10) vs 19% (n = 7) (*P* = .41), and 5% (n = 1) vs 5% (n = 1) (*P* = .95), respectively. Similar results were seen in the PP population. In Figure 2 we present a Kaplan-Maier plot of overall survival demonstrating no significant difference between groups. Median survival was 246 days (95% CI, 163-307 days) with SOC and 164 days (95% CI, 117-278 days) with SOC plus disulfiram and copper. The results from PP analyses (eTable 1, eTable 2, and eFigure in Supplement 2), including between-group comparisons, did not differ from the intention-to-treat analyses.

### Secondary Outcomes

Several secondary end points, such as PFS, HRQoL, and volumetric expansion, are summarized in Table 3. There were no significant between-group differences. Median PFS was similar in the 2 groups with 2.6 months (95% CI, 2.4-4.6 months) after SOC and 2.3 months (95% CI, 1.7-2.6 months) with SOC plus disulfiram and copper. The EQ-5D scores were similar in the 2 groups at baseline and during follow-up at 3 months and 6 months, but fewer patients in the SOC plus disulfiram and copper group completed the HRQoL questionnaires during follow up (23% [10 of 43] of patients in the SOC plus disulfiram and copper group completed the HRQoL questionnaires at 3 months vs 42% [19 of 45] in the SOC group). We had 13 images to evaluate in the SOC group and 13 in the experimental group. The mean (SD) daily growth based upon volumetric analyses were not significantly different between groups, with 12% (23%) volumetric expansion in the SOC group and 3% (8%) in the SOC plus disulfiram and copper group (*P* = .19).

**Table 3.** Secondary Outcomes Other Than Survival in the Intention-to-Treat Population

Intention-to-treat population	SOC (n = 45)	SOC plus disulfiram and copper (n = 43)	*P* value
PFS, median (95% CI), d	77 (73-138)	68 (50-78)	.07
Progression at 6 mos			
No./total No. (%)	32/42 (76)	35/43 (81)	.56
Progression at 12 mos			
No./total No. (%)	32/37 (86)	36/37 (97)	.09
Change in EQ-5D index value from baseline to 3 mos			
No.	19	10	.99
Mean (SD)	−0.04 (0.18)	−0.04 (0.17)	
Change in EQ-5D index value from baseline to 6 mos			
No.	12	4	.46
Mean (SD)	−0.1 (0.24)	−0.003 (0.22)	
Daily change in tumor volume			
No.	13	13	.19
Mean (SD), %	12 (23)	3 (8)	

Abbreviations: EQ-5D, EuroQol-5D-3L; PFS, progression-free survival; SOC, standard-of-care alkylating chemotherapy.

### Safety

The safety population was used in the safety analyses. In the treatment group with SOC plus disulfiram and copper, there were significantly more patients with adverse events (AE) CTCAE grade 3 or higher (14 patients [34%] vs SOC group: 5 [11%]; *P* = .02) or any SAE (17 patients [41%] vs SOC group: 7 [16%]; *P* = .02) (eTable 3 in Supplement 2). Six patients (15%) in the experimental group developed elevated liver enzymes compared with no patient in the SOC group. One fatal SAE was reported in the SOC plus disulfiram and copper group. This event was a hemorrhage in a progressive tumor and was not considered related to the treatment. Nine patients (22%) in the group receiving SOC plus disulfiram and copper experienced at least 1 SAE with probable, possible, or definite relationship to the intervention, as assessed by the treating clinician and/or a clinical pharmacist.

## Discussion

In this open-labeled, multicenter, randomized clinical trial there was no significant survival benefit of SOC plus disulfiram and copper compared with SOC in patients with recurrent GB. No significant between-group difference was seen in HRQoL. There were significantly more AEs and SAEs in patients treated with SOC plus disulfiram and copper. Despite very promising preclinical reports, disulfiram and copper does not have a clinical benefit in patients with recurrent GB.

Our results are in accordance with the previous clinical studies of disulfiram in patients with GB.^22,29,30^ In a phase 2, open-label single-group study of disulfiram (and copper) 80 mg 3 times daily demonstrated no objective responses and a median survival of 7.1 months,^30^ compared with 5.5 months in our trial, and both compared unfavorably with lomustine in landmark studies in recurrent GB.^31,32^ On the other hand, our control group with SOC, where lomustine was used in the majority of cases, had results in line with these studies (median overall survival: 8.2 months [95% CI, 5.4-10.2 months]).

We were not able to translate the numerous encouraging preclinical results to the clinical setting.^4,8,13,14,15,16^ Several studies have indicated a synergistic effect between temozolomide (or other alkylating agents) and disulfiram,^8,16,33^ although 1 study found that temozolomide somehow antagonized the effects of disulfiram.^34^ It has been suggested that disulfiram-copper induce MGMT inhibition, but also increased replication stress and DNA damage, hence there are arguments that SOC plus disulfiram and copper could potentiate treatment effects, both for patients with unmethylated and with hypermethylated MGMT promoter.^8,16,19,35^ The complete lack of positive signal in our trial indicate that this has no clinical relevance in patients with recurrent GB.

Recent preclinical data suggest lack of therapeutic effect of disulfiram if there is interference by cannabidiol and related drugs, not infrequently used by patients to mitigate pain. Cannabidiol induces expression of metallothioneins that bind CuET, the active copper-containing anticancer metabolite of disulfiram, thereby undermining the antitumoral effect of disulfiram.^36^ However, since the use of cannabis is illegal in Sweden and Norway, we do not believe that such interference is a likely explanation for the negative outcome of our study.

Currently it is difficult to envision a future role of disulfiram and copper in treatment of GB, although new combinations and potential clinical useful synergies with other treatments cannot be ruled out. Concerning combined treatments, disulfiram is “the backbone” of the CUSP9 regimen which seems theoretically intriguing but to our knowledge this concept still lacks clinical evidence.^33,37,38^

We considered the reports of tolerability of disulfiram in range 250 to 500 mg daily.^22,37,39^ Still, we experienced substantial concern with disulfiram 400 mg daily with patients experiencing more AEs and SAEs. A prior study in patients with prostate cancer suggested poor tolerability of disulfiram dosage 500 mg daily, but better tolerability with 250 mg.^40^ Disulfiram 250 mg daily in addition to copper supplement was well tolerated in patients with advanced solid tumors including liver involvement, although no tumor response was noted.^41^ The final report by Huang and colleagues in patients with GB suggested that 15% treated with 500 mg daily had dose-limiting toxic effects, whereas in their phase 2 study the regimen of 80 mg 3 times daily was well tolerated.^29,30^ These experiences are necessary for clinical researchers to take into account before attempting to repurpose disulfiram in anticancer treatment.

A possible reason why we failed in translating the laboratory results to the clinic may be inadequate bioavailability in the target tissue. We do not know the tissue concentrations reached in the present study, but 400 mg daily (allowing 200 mg in case of toxicity) could be considered sufficient given the signal in a study using 40 mg 3 times daily in patients with lung cancer.^20^ Nevertheless, from a toxicity point of view, a higher dose of disulfiram is not feasible. Except from the small study in non–small cell lung cancer, the positive laboratory findings have been difficult to translate to clinical benefit also for other cancers, with negative trials in patients with prostate cancer and in cisplatin-responsive malignant neoplasms.^21,40,42,43^

Considering the difficulties in translating the promising preclinical work to clinical benefit, future work needs to focus on novel application methods or treatment synergies, and some novel strategies have recently been published.^44,45^ Disulfiram may also play a role as radiosensitizer and may have a clinical benefit under different circumstances than provided in our trial.^17,18^

### Limitations

The study has several limitations. First, the study did not reach the intended power as the interim analysis demonstrated more SAEs combined with futility in the experimental group.^46^ Second, the open-label design of our trial carries an inherent risk of bias among the investigators in the reporting of AEs and progressive disease. However, this possible bias would be unlikely to have affected the primary outcome survival. Third, it is possible that some patients in the experimental group terminated chemotherapy earlier due to toxic effects from disulfiram, but the most common reason for discontinuation of chemotherapy was progressive disease. Fourth, for HRQoL, we had limited data, especially in the SOC plus disulfiram and copper group, as few patients completed the questionnaires during follow-up.

## Conclusions

This randomized clinical trial found that the addition of disulfiram and copper to alkylating chemotherapy did not improve survival in patients with recurrent GB. Instead, the treatment regimen of 400 mg disulfiram daily resulted in significantly more toxic effects. These results suggest that disulfiram and copper is not of benefit in patients with recurrent GB.

## Notes

Supplement 1.
Trial Protocol and Statistical Analysis Plan

Supplement 2. **eTable 1.** Baseline Characteristics in the Per-Protocol Population **eTable 2.** Treatment Characteristics in the Per-Protocol Population **eFigure.** Overall Survival in the Per-Protocol Population **eTable 3.** Summary of Adverse Events in the Safety Population

Supplement 3.
Data Sharing Statement
