# Supplementary material for: Effect of Disulfiram and Copper Plus Chemotherapy vs Chemotherapy Alone on Survival in Patients With Recurrent Glioblastoma: A Randomized Clinical Trial
Source: JAMA Netw Open. 2023 Mar 31;6(3):e234149. doi: 10.1001/jamanetworkopen.2023.4149 (PMC10066460; doi:10.1001/jamanetworkopen.2023.4149)
Supplement: Supplement 1. — Trial Protocol and Statistical Analysis Plan [file jamanetwopen-e234149-s001.pdf]

<table>
<tr><td rowspan="3">PROTOCOL:</td><td>ETHICAL REFERENCE:</td><td>Norway: 2016/283 (approved)
Sweden: Dnr 332-16 (approved)</td></tr>
<tr><td>SWEDISH MEDICAL PRODUCTS AGENCY REFERENCE</td><td>5.1-2016-25235 (approved)</td></tr>
<tr><td>NORWEGIAN MEDICINES AGENCY REFERENCE:</td><td>16/04133 (approved)</td></tr>
<tr><td rowspan="5">DIRECT (DIsulfiram REsponse as add-on to ChemoTherapy in recurrent) Glioblastoma: A randomized controlled trial</td><td>CLINICALTRIALS NR:</td><td>NCT02678975</td></tr>
<tr><td>EUDRACT NR:</td><td>2016-000167-16</td></tr>
<tr><td>DATE</td><td>18th Feb 2019</td></tr>
<tr><td>VERSION:</td><td>Version 2.1</td></tr>
<tr><td>SPONSOR:</td><td>Department of Neurosurgery, Sahlgrenska University Hospital with principal investigator Asgeir S. Jakola</td></tr>
</table>

Clinical trial protocol DIRECT glioblastoma

Trial type	Multicenter, open labeled randomized controlled trial with parallel group design (1:1)
Clinical phase	Medical intervention corresponding to a phase II/III trial
Short title	DIRECT glioblastoma
Full title	Disulfiram response as add-on to chemotherapy in recurrent glioblastoma: A randomized controlled trial
Registered	EudraCT and Clinicaltrials.gov
Sponsor	Sahlgrenska University Hospital
Sponsor-investigator	Asgeir S. Jakola, Department of Neurosurgery, MD, PhD
Trial coordinator	Asgeir S. Jakola, MD, PhD
Safety reporting	Läkemedelsenheten, Klinisk Farmakologi, Sahlgrenska Universitetssjukhuset.<br>e-mail: biverkningsenheten@vgregion.se<br>fax: +46 (0)31 41 01 42<br>cc e-mail: Asgeir.jakola@vgregion.se
Clinical condition	Recurrence or progression of glioblastoma
Investigational product	Disulfiram (Antabus®) together with copper supplement
Trial intervention	Disulfiram 400 mg once daily in addition to copper supplement (2 mg once daily) and alkylating chemotherapy
Control group intervention	Alkylating chemotherapy

Sample size	Assuming an improvement in proportion achieving 6-month survival from 60 % to 80 % we need a sample size of 128 in total (64 in each group; alpha 0.1, power 80 % and two sided test). *With expected 10 % attrition this equals 142 (71 in each group) included patients.*
Trial duration	Four to five years
Planned recruitment	Autumn 2016 to Spring 2021
Participating sites	Oncological units in Sweden and Norway
Responsible for randomization	Applied Clinical Research,<br>Norwegian University of Science and Technology, Trondheim, Norway
Monitoring	Norway: Applied Clinical Research, Norwegian University of Science and Technology, Trondheim, Norway<br>Sweden: Klinisk prövningsenhet, Verksamhet onkologi, Blå Stråket 2, Sahlgrenska Universitetssjukhuset, 413 45 Göteborg, Sweden

**BACKGROUND**	Disulfiram (Antabus®) is a well-tolerated, cheap, generic drug that has been in use since the 1950s to treat alcoholism. There is now an increasing amount of independent preclinical data to support disulfiram as an anticancer agent. In glioblastomas add-on treatment to alkylating agents may offer additional benefit due to O^6-methylguanine methyltransferase (MGMT, a DNA-repair enzyme) inhibition. The potency of disulfiram as an anticancer agent seems strengthened by copper. There is now anecdotal clinical evidence of disulfiram as an anticancer, for instance with improved survival in lung cancer patients. Also, disulfiram was positively associated with survival in a registry study. Collectively these results indicate a clinical meaningful response. So far no clinical studies have been published in glioma patients, but two small, uncontrolled studies are planned according to clinicaltrials.gov. with search 1st November 2015.
**OBJECTIVES**	To investigate disulfiram and copper-supplement as add-on treatment in glioblastoma patients receiving alkylating chemotherapy. This will to our knowledge be the first planned RCT with disulfiram in glioblastoma patients, and will consequently serve as a proof-of concept study.
**PRIMARY OUTCOME**	Survival at 6 months
**SECONDARY OUTCOMES**	- Actual survival analyzed at 9, 12 and 24 months.<br>- Median progression free survival<br>- 6 and 12 month progression free survival<br>- Median overall survival<br>- Health-related quality of life<br>- Volumetric expansion<br>- Toxicity/safety
**KEY INCLUSION AND EXCLUSION CRITERIA**	<u>Key inclusion criteria</u><br>**1.** A previous diagnosis of glioblastoma (histologically verified) and presenting with a first progression/recurrence documented by MRI.<br>**2.** Indication for treatment with chemotherapeutic alkylating agents (i.e. temozolomide OR lomustine (also known as CCNU) including the so called PCV treatment (procarbazine, CCNU,

	vincristine)).<br>**3.** Age 18 years or older.<br>**4.** Karnofsky performance status of 60 – 100<br>**5.** Willing to refrain from ingestion of alcoholic beverages<br><br><u>Key exclusion criteria</u><br>**1.** Earlier chemotherapy for progression<br>**2.** Received radiotherapy within the 3 months before the diagnosis of progression<br>**3.** History of idiopathic seizure disorder, psychosis or schizophrenia.<br>**4.** History of uncontrolled hypertension (i.e. systolic BP > 180 mmHg) or a diagnosis of congestive heart failure<br>**6.** History of active liver disease<br>**5.** History of Wilson's disease or family member with Wilson's disease (unless excluded as a carrier by genetic test).<br>**6.** Use of medications such as metronidazole, warfarin, theophylline, phenytoin, phenobarbital, chlordiazepoxide, imipramine, diazepam, isoniazid, amitriptyline within 14 days prior to the first dose of disulfiram. *Of note, lorazepam and oxazepam are not affected by the P450 system* and are not contraindicated with disulfiram).<br>**7.** Unfit for participation for any other reason judged by the physician including patients
**FUNDING**	Nordic Cancer Union, AG foundation, ALF agreement, SU foundation, Swedish Cancer Foundation

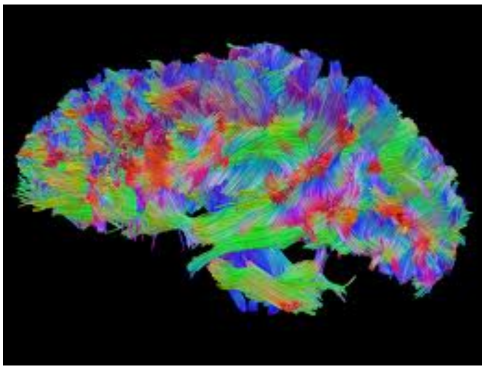

Content

1. Background **6**
1.1. The case for Disulfiram (Antabus®) *6*
1.1.1. Generic drug with well-known safety profile 6
1.1.2. Preclinical evidence 7
1.1.3. Clinical evidence to date 7
1.1.4. Dose-rationale 7
1.1.5. Explanation for choice of comparator 8
1.1.6. Justification of choice of trial population 8
1.1.7. Risk and benefit 8

2. Project aim **9**

3. Methods **9**
3.1. Primary end-point *9*
3.2. Secondary end-points *9*
3.3. Imaging *9*
3.4. Patient reported outcomes *10*
3.5. Power estimation *10*
3.6. Sample size *10*
3.7. Inclusion criteria *10*
3.8. Exclusion criteria *11*
3.9. Informed consent *12*
3.10. Handling of the experimental drug and the nutritional supplement *12*
3.11. Data Collection *12*
3.11.1. Blood samples 12
3.11.2. In case of temozolomide treatment 12
3.11.3. In case of lomustine/PCV treatment 12
3.11.4. Delay in treatment 12
3.12. Compliance, cross-over and individual stopping rules *13*
3.12.1 Compliance 13
3.12.2. Modification in disulfiram treatment before progression or in case of glioblastoma progression 13
3.12.3 Cross-over 13
3.12.4 Discontinuation/stopping-rules 13
3.13. Interim analysis and abortion criteria 14
3.14. Methods of minimizing bias 14
3.14.1. Randomization 14
3.14.2. Blinding procedures 14
3.14.3. Other methods 14

4. Study schedule **14**

5. Safety **15**
5.1. General remark on safety of interventional treatment *15*
5.2. Definition adverse event *16*
5.3. Toxicity *16*
5.4. A Serious Adverse Event (SAE) *16*
5.5. Serious Adverse Reaction (SAR) and Suspected Unexpected Serious Adverse Reactions (SUSAR) *17*
5.6 Special considerations for women of childbearing potential *18*

6. Trial conduct **18**

7. Quality assurance and control **18**
7.1. Case report forms *18*

7.2. Patient security 18
7.3. Specification of source documents 18
7.4. Archiving 18
7.5. Data management 19
7.6. Analysis 19
7.7. Monitoring 19
7.8. Inspection 19
7.9. Premature Termination of the Study 19

8. Insurance 20

9. Regulatory requirements 20

10. Cost 20

11. Estimated progress 20

12. Contact information 21
12.1. Sponsor/principal investigator: 21
12.2. Monitor 21
12.3. On-site responsible physicians/investigators 21

13. References 21

14. Signature pages 24
Sponsor and co-ordinating investigator 24
On-site responsible physicians/investigators 25

15. Attachments 32

1. Background

Gliomas are the most common malignant brain tumor, and they result in more years of life lost than any other tumor group.[1] The annual age adjusted incidence rate is approximately 9 per 100,000 in adults.[1] Glioblastoma, WHO grade IV astrocytoma, is the most common subtype and has a poor survival with median survival of only 10 months.[2] Extensive surgical resections followed by postoperative fractioned radiotherapy and concomitant and adjuvant temozolomide (an alkylating agent) prolong survival in glioblastoma (WHO IV) and is the mainstay of treatment.[3] Still, the median overall survival in the RCT by Stupp et al. was only 14.6 months. The effect of temozolomide seems at least in part dependent of O^6-methylguanine methyltransferase methylation (MGMT, a DNA repair enzyme), leading to impaired DNA repair of damages caused by temozolomide within the tumor.[4] Unfortunately less than half of glioblastoma patients harbor MGMT methylation.[4]

In the recurrent situation there is no true consensus on what to use. A common approach is to prescribe temozolomide again as long as the progression occurs more than 3-6 months after ended primary treatment, and especially if initial response was observed. However, in cases where progression is seen on ongoing or early after temozolomide treatment it is common practice to prescribe lomustine (also known as CCNU)[5] or PCV (procarbazine, CCNU, vincristine). Still, the treatment effect in this situation is modest and new treatments are clearly indicated.

Based on knowledge of pathophysiology and pharmacology, the 'repurposing' or 'repositioning' of approved drugs as treatments for other diseases is currently a topical issue relevant for industry, academia and governmental policies. According to Francis Collins, the Director of the National Institutes of Health, research in this direction should be 'an important focus of the NIH's proposed National Center for Advancing Translational Sciences'.[6]

1.1. The case for Disulfiram (Antabus®)

1.1.1. Generic drug with well-known safety profile

Disulfiram is a cheap, generic drug that has been used since 1947 to treat alcoholism and is most commonly known as "Antabus®".[7] The drug is well tolerated (as long as one avoids alcohol), however some caution is necessary with respect to comorbidities and interactions. Health authorities (i.e. EMA and FDA) have already approved its use in humans, but only for the indication alcohol dependency.

Disulfiram safety is well documented. In alcohol dependent patients long-term use (> 1 year) has also been demonstrated to be safe and even normalization of liver enzymes initially elevated due to alcohol use has been observed during disulfiram treatment.[7,8] However, there are reports that in patients with alcohol dependence disulfiram may cause hepatitis in 1:30.000 cases.[7] There are also reported cases of neuropathy with a frequency of 1:15.000 treatment years, with typically dose-dependent occurrence with doses of > 500 mg/day and long-term treatment with the typical presentation after 1 year of treatment.[7] Optic neuritis may be more frequent in smokers, thus it may be worthwhile informing patients to quit before starting treatment (www.fass.se). However, neuropathy including optic neuritis is typically reversible if detected early. Psychosis and other acute confusional states beginning with fatigue and forgetfulness, rarely proceeding to ataxia or stupor has been reported, but psychiatric syndromes are common in alcohol dependent patients, and in a controlled study

there were no differences in confusion/psychosis between groups receiving disulfiram or not.[7] Nevertheless, confusion appear to have occurred more often earlier when dosage regimens typically were > 500 mg/day. Because of the potentially fatal outcome of a disulfiram-alcohol reaction in a patient with heart disease disulfiram should normally not be offered patients with impaired cardiac function (i.e. heart failure). Still, the most important issue is total abstinence from alcohol.[7]

In our context with cancer patients in need of chemotherapy, recent studies with disulfiram (or its metabolite) as add-on report minimal toxicities.[9,10] The combination of chemotherapy and disulfiram in preexisting disulfiram users developing cancer is a common clinical practice. And a phase I study on disulfiram in glioblastoma patients taking temozolomide was well tolerated below 500 mg daily dose.

1.1.2. Preclinical evidence

Disulfiram has demonstrated a wide range of antineoplastic effects with potential effect in gliomas and this has been translated into promising effects in cell lines and cell cultures.[11-17] The anti-cancerous effect has also been demonstrated in different, independent animal models.[11,17] It is now suggested that disulfiram should be implemented in clinical trials[18] and indeed 2 small non-randomized studies are registered in clinicaltrials.gov when searching "glioma" AND "disulfiram" (www.clinicaltrials.gov, search date 01th November 2015). The drug may further potentiate alkylating damage possibly through inhibition of MGMT.[19]

1.1.3. Clinical evidence to date

Dsiulfiram-copper complex (Cu-DSF) has been considered to be the (most) active antineoplastic metabolite of disulfiram in terms of cancer cells toxicity and ubiquitin proteasome system (UPS) interference.

In two small RCTs, one on breast cancer (64 patients) and one in metastatic lung cancer disulfiram (or one of its metabolites) has been tested with promising results on overall survival.[9,10] Also, in a small phase I study in glioblastoma promising results were observed.[20]

1.1.4. Dose-rationale

The administration of treatment with disulfiram and copper is scheduled to start concomitant with alkylating chemotherapeutic treatment. The administration of disulfiram and copper supplement should be continued as adjuvant after chemotherapy withdrawal with continued administration as long as tumor targeted therapy is indicated.

Disulfiram:

Disulfiram as an anti-cancer agent has been suggested at a dose of 250 mg twice daily[18] although the pill in common in Norway and Sweden is 200 mg. A daily dose > 500 mg/day of disulfiram is associated with adverse-effects. Disulfiram is cheap, with a cost of administration of 400 mg a day below both 10 NOK and SEK.

Patients will take disulfiram (Antabus®) as a once daily dose of 400 mg and this decision is based on the toxicity profile of the drug. This dose is also within the accepted regimen to treat alcoholism. In case of intolerance, lower dose of 200 mg per day is allowed. Patients will take disulfiram in the evening. Patients have to avoid alcohol.

Copper:

Copper supplementation will be given separately from disulfiram (in the morning). Copper nutritional supplement will correspond to 2,5 mg of elementary copper and this is not

considered a safety concern[21]. This dose is within the typical copper intake in western countries. WHO has concluded that the fatal oral dose of copper salts is about 200 mg/kg body weight.[21]
Thus, the nutritional supplementation of copper in this study is to ensure that copper levels are adequate to keep copper homeostasis with access to generate the Cu-DTC complex also in a patient population where dietary intake may be insufficient.

Concerning chronic toxicity there were no reported problems when the drinking water in in areas over time contained copper at concentration of 8 mg/L.[21] Nevertheless, potential adverse reaction of copper intoxication includes salivation, epigastric pain, nausea, vomiting, diarrhea, renal and hepatic injury.

We will provide the nutritional supplement of copper through a product called Chelated Copper by Solgar® sold at various stores specialized in "health foods" and "dietary supplement health". Examples are:

a) Sweden, 2,5 mg copper: http://www.lifebutiken.se/produkter/vitaminer-mineraler/ovriga-vitaminer-mineraler/kelaterad-koppar-2-5-mg
b) Norway, 2,5 mg copper: http://www.life.no/produkter/vitaminer-mineraler/ovrige/chelated-copper-100-tabl

1.1.5. Explanation for choice of comparator

In the recurrent situation there is no true consensus on what to use. A common approach is to prescribe temozolomide again as long as the progression occurs more than 3-6 months after ended primary treatment, and especially if initial response was observed. However, in cases where progression is seen on ongoing or early after temozolomide treatment it is common practice to prescribe lomustine (also known as CCNU)[5] or PCV (procarbazine, CCNU, vincristine). Lomustine is also the suggested control arm in studies in recurrent glioblastomas due to its established effect in this phase.[22] The downside is hematological toxicities, especially in patients previously treated with temozolomide.[22] Both abovementioned standard treatments have very modest effect in the recurrent setting and new treatments are clearly indicated.

Also MGMT inhibition is seen with disulfiram in preclinical data, and this is predictive of response to alkylating agents.

Consequently, we have chosen any alkylating chemotherapy as the control arm where the intervention groups receive disulfiram and copper supplement given as add-on to chemotherapy.

1.1.6. Justification of choice of trial population

Glioblastoma in the recurrent situation is very treatment resistant and is an immediate threat to patients' survival. No true consensus exists on treatment in the recurrent situation. Clearly, in this situation experimental therapy is justified and especially one with expected low toxicity such as disulfiram hopefully not having serious detrimental effects on quality of survival.

1.1.7. Risk and benefit

It may be a personal sacrifice for patients avoid alcohol intake completely. Further, disulfiram has a well-known risk profile and the major risks of treatment with 400 mg disulfiram daily lie mainly in lack of compliance with respect to alcohol intake and the rare, but potentially

serious hepatitis. The risks, also together with chemotherapy in this context, are therefore limited.[20] In addition, there is a risk of neuropathy that is reversible upon drug discontinuation.[20] The potential benefit lies in improved response to treatment. It is the sponsor´s impression that disulfiram treatment compared to regular chemotherapeutic agents has a favorable risk profile and that pursuing clinical studies to elucidate on treatment efficacy is warranted. The sponsor thus concludes that the overall potential benefit clearly outweighs the risks associated with treatment.

2. Project aim

The project is planned for 6 years and with the following aim:

Gaining initial clinical experience of disulfiram efficacy as an active therapy for human glioblastoma, performing the first randomized controlled study with this treatment.

This will be accomplished in a clinical multicenter study carried out at academic hospitals in Sweden and Norway.

3. Methods

This trial is a multicenter two-armed open labeled 1:1 randomized controlled trial. The study will be conducted in several Swedish and Norwegian oncological units..

3.1. Primary end-point

Survival at 6-months analyzed according to intention-to-treat.

3.2. Secondary end-points

a) Actual survival analyzed at 9, 12 and 24 months.
b) Median progression free survival
c) 6 and 12 month progression free survival
d) Median overall survival
e) Health-related quality of life[23] (see attachment 1)
f) Volumetric expansion[24]
g) Safety assessment reporting the proportion of grade 3 and 4 adverse effects Toxicities were assessed with the Common Terminology Criteria for Adverse Events (CTCAE) version 4.0 (see attachment 2)

For evaluation of end-points, see attachment 3.
In general, all analyzes will be performed according to intention-to-treat (primary aim) and explored as as-treated analyses. All endpoints will be analyzed with an investigator blinded for the treatment arm. Concerning the primary end-point we do not expect any missing data.

3.3. Imaging

MRI according to clinical protocol, including preoperative and postoperative MRI if undergoing repeated resection and with a new MRI scan routinely scheduled every 3-month thereafter up to 24 months post-inclusion. This should be implemented also after documented

progression in the disulfiram group if patient continues on disulfiram alone or in combination with another rescue therapy and in the control group as long as tumor targeted therapy is given.

3.4. Patient reported outcomes

EQ-5D 3L monitored every 3-month, continued until progression, death or 24 months post-inclusion (EQ-5D 3L; see attachment 1).

3.5. Power estimation

The analysis below is based on data from the BELOB trial (median in lomustine group; 1 month (95 % CI 1-3 months); 13 % PFS at 6 month; median overall survival 8 months (95 % CI 6-11); 30 % overall survival at 12 months).[5] We will use 6 month survival (analyzed at 6 months post-randomization of the last patient included) as primary end-point, but relaxing the alpha value to 0.10 (by convention 0.05) due to the phase II setting.[25] As stated by Khan et al; phase II studies are "preliminary assessment of a new intervention before embarking on a larger and expensive randomized controlled trial",[25] thus slightly increasing the chance of false positive results (due to chance).

3.6. Sample size

Based on the assumption that the treatment group will have an improvement in proportion achieving 6-month survival from 60 % to 80 % we need a final sample size of 128 in total (64 in each group; alpha 0.1, power 80 % and two sided test, see below). Considering 10 % attrition the actual included sample need to be **142 with 71 patients included in each group.**

Sample size (without continuity correction)			
	N	Alive	Dead
disulfiram	64	51	13
controls	64	38	26
TOTAL	128	89	39

http://www.sample-size.net/sample-size-proportions/
alpha 0.1
two sided test
power 80 %

RR: 1.3333
Risk 60 % of death within 6 months versus 80 % risk of death within 6 months

3.7. Inclusion criteria

The subjects must fulfill all the following inclusion criteria to be eligible for participation in the study, unless otherwise specified:

1. A previous diagnosis of glioblastoma/gliosarcoma (later referred only to glioblastoma) that was histopathologically verified at time of diagnosis, now presenting with a first progression/recurrence documented by MRI (see attachment 4).
2. Indication for treatment with chemotherapeutic alkylating agents (e.g. temozolomide OR lomustine including PCV treatment).
3. Age 18 years or older.
4. Karnofsky performance status of 60 – 100 (see attachment 5).

5. Not receiving another experimental treatment, including compassionate use programs, for glioblastoma at the moment of inclusion or during active treatment within the assigned group (i.e. control or disulfiram group).
6. Able to take oral medications.
7. No known allergy to disulfiram or copper.
8. Absolute neutrophil count ≥ 1,500/mcL and platelets ≥ 100,000/mcL
9. Willing to refrain from ingestion of alcoholic beverages while on the study is a criteria to be randomized. However, once randomized alcohol abstinence only affects the group treated with disulfiram, and in this group it includes the entire period and one month after last dosage of disulfiram.

3.8. Exclusion criteria

Potential study subjects who meet any of the following criteria are not eligible for participation in the study:

1. Earlier chemotherapy for progression (e.g. "rescue therapy")
2. History of idiopathic seizure disorder, psychosis or schizophrenia.
3. History of uncontrolled hypertension (i.e. systolic BP > 180 mmHg) and a diagnosis of congestive heart failure
4. Received radiotherapy within the 3 months before the diagnosis of progression (see also attachment 4).
5. Addiction to alcohol or drugs.
6. Pregnant and/or breastfeeding.
7. Women of childbearing potential who do not have a negative pregnancy test taken no longer than 14 days prior to randomization.
8. History of active liver disease, including chronic active hepatitis, viral hepatitis (hepatitis B, C and CMV), cholestatic jaundice of any etiology or toxic hepatitis or inadequate hepatic function, defined as baseline ASAT and ALAT > 2.5 X upper institutional limit and/or bilirubin > 2.0 X upper institutional limit.
9. History of Wilson's disease or family member with Wilson's disease (unless excluded as a carrier by genetic test).
10. History of hemochromatosis or family member with hemochromatosis (unless excluded as a carrier by genetic test).
11. Nickel hypersensitivity (disulfiram mobilize nickel causing a brief increase in nickel concentrations before excretion. The initial increase may lead to hepatitis in predisposed patients).[7]
12. Need for metronidazole, warfarin and/or theophylline medication (the metabolism may be influenced by disulfiram).
13. Patients who are taking medications metabolized by cytochrome P450 2E1, including chlorzoxazone or halothane and its derivatives (phenytoin, phenobarbital, chlordiazepoxide, imipramine, diazepam, isoniazid, metronidazole, warfarin, amitriptyline within 14 days prior to the first dose of disulfiram. *Of note, lorazepam and oxazepam are not affected by the P450 system* and are not contraindicated with disulfiram).
14. Unfit for participation for any other reason judged by the including physician.

3.9. Informed consent

Patients will be included only after the informed consent is signed (see attachment 6a: Swedish and 6b: Norwegian). The informed consent needs to be dated at time of inclusion, be explicit and written and may at any time be withdrawn by the participant. Patients will be asked to participate during an outpatient visit to the local neurologist or oncologist. Also, the right of the participant to refuse to participate without giving reasons must be respected.

Patients without possibility to provide written, informed consent (e.g. severe cognitive deficit and aphasia) are not to be included via proxies.

3.10. Handling of the experimental drug and the nutritional supplement

The experimental therapy consists of disulfiram treatment and nutritional copper supplement. In Norway and Sweden, copper supplement will be delivered to study participants randomized to the experimental arm by the local investigator. Based on the safety profile and long experience with the experimental drug the local investigator prescribes Disulfiram, and the necessary information will be included in the prescription. Patients will collect disulfiram at the pharmacy similar to any other prescription. In Sweden due to a reimbursement arrangement patients will not experience extra expenses ("högkostnadsskyddet"). Norwegian sites will also prescribe the drug from the pharmacy and the batch number of disulfiram will be registered by the pharmacy in a log available also to the local investigator upon request. After agreement with the local hospital pharmacy the cost of the drug will be charged the sponsor directly.

3.11. Data Collection

Data will be recorded in web-based case report forms (eCRFs). A data collection manual will be supplied to all centers and each center is responsible to know the content and the routines for all data collection.

All CRFs will be accessible by sponsor to assess data entry, review of drug safety and ultimately, statistical analysis.

3.11.1. Blood samples

Blood samples to be samples are consider clinical routine and taken at routine intervals: Hemoglobin, thrombocytes, leukocytes with differential count, ASAT, ALAT, GT, ALP, bilirubin and INR.

3.11.2. In case of temozolomide treatment

In these cases we plan assessment every 4 weeks to coincide with clinical routine.

3.11.3. In case of lomustine/PCV treatment

There will not be monthly follow up, but at every 6 weeks to follow the clinical routine. We will register the first control at 1 month, then at 3 month, 4 and 6 months and so on. This is chosen to minimize patient burden and may lead to 4 more observations in the temozolomide group than in the lomustine group. Still, the visit planned every third month with MRI is identical.

3.11.4. Delay in treatment

In case of co-morbid events leading to delay or breaks in treatment the assigned treatment is started when appropriated as judged by the local physician. The patient is entered into the study schedule at the nearest time-point to actual treatment with no additional visits planned

to correct for this to minimize patient burden. Further entry into study then follows the study schedule.

3.12. Compliance, cross-over and individual stopping rules

3.12.1 Compliance

Compliance of disulfiram and copper treatment will be assessed by tablet count at controls in addition to patient self-reporting. If less than 50 % of expected tablets are consumed the patient will be considered non-compliant (in the given period), and if the patient at follow-up visit then wish to withdraw the patient will be regarded as cross-over from last visit (including non-compliant period). This is for descriptive analyses, or for post hoc analyzes, since the pre-defined end-points are analyzed according to intention-to-treat.

3.12.2. Modification in disulfiram treatment before progression or in case of glioblastoma progression

Treatment with disulfiram is continued as long as any tumor targeting treatment is indicated, (i.e. until patient moves into strict palliative phase).

- In case of discontinuation of alkylating chemotherapy before any signs of progression (e.g. due to adverse events specific to chemotherapy including hematological toxicities) treatment with disulfiram is continued either as a single agent or combined with another chemotherapy as long as this is outside of a clinical trial. In fact, the treating oncologist is encouraged to combine further disulfiram treatment with another second line rescue therapy (at the discretion of the treating physician).
- If progression is documented, change of chemotherapy may occur. In case of prescription of new cytotoxic treatment (2nd line rescue therapy), disulfiram should be continued in the group randomized to disulfiram treatment.

In all cases where disulfiram is continued, but with modifications from the original assigned treatment, the patient should be followed with visits, imaging and blood samples according to the protocol.

3.12.3 Cross-over

Before study closure *no crossover from the control group to disulfiram is allowed.*

3.12.4 Discontinuation/stopping-rules

Thus, treatment with disulfiram is only to be discontinued when:

- Unacceptable adverse effect(s) as judged by the treating doctor
- No longer indication for tumor targeting therapy
- The patient wishes to withdraw from treatment
- In case of certain grade 3 and 4 adverse events believed to be caused by disulfiram treatment or put patients on disulfiram treatment to excess risk (see below).
- Other

Discontinuation criteria for disulfiram at the individual level due to adverse effect(s)

- Hepatobiliary adverse effect grade 3 and 4.
- Development of congestive heart failure grade 3 and 4.
- 2 or more hospitalizations due to status epilepticus (i.e. grade 4 adverse event).
- New onset delirium and/or psychosis grade 3 or 4 adverse effect not induced by increasing dose of corticosteroids.

- Other grade 3 or 4 adverse effects where treating physician recommend discontinuation of disulfiram

Reasons for discontinuation of assigned treatment (control group)
- Unacceptable adverse effect(s) as judged by the treating doctor
- No longer indication for tumor targeting therapy
- The patient wishes to withdraw from treatment
- Other

3.13. Interim analysis and abortion criteria

At 50 % inclusion (71 included patients) an interim analysis will be performed. In this analysis serious adverse events will be explored. A data monitoring scientific committee (DMSC) independent of sponsor and local investigators will be set up to review confidentially the results of this interim analysis (see attachment 7).

3.14. Methods of minimizing bias

3.14.1. Randomization

This is an open-label trial (i.e. no placebo) with alkylating chemotherapy in the group randomized to control and alkylating chemotherapy with add-on of disulfiram in the group randomized to intervention.

Patients will be randomized separately at each study center. Blocks of varying sizes will be used to make prediction of allocation impossible.
Randomization will be performed by a web-based randomization system developed and administered by Unit for Applied Clinical Research, Norwegian University of Science and Technology.

3.14.2. Blinding procedures

The trial is open labeled; however a statistician blinded for treatment assignment will perform data analyses. A radiologist blinded for treatment allocation will evaluate radiological end-points.

3.14.3. Other methods

We will not stratify for more than study center, however important base-line variables like repeated surgery, time since primary treatment, age, functional status, lomustine versus temozolomide, biomarker status from initial surgery if available (IDH and MGMT) will be registered and adjusted for using post-hoc exploratory analyses in case of skewed randomization.

4. Study schedule

As seen from the study schedule the patients are in the trial follow-up for 24 months. Earlier reason for discontinuation of follow-up includes death or any criteria mentioned in the 3.11.4 section above. Even though the patients return to current clinical practice after 3.11.4 discontinuation, we will register progression and survival since they are main study outcomes. To minimize patient burden in case of treatment discontinuation we will not continue to measure quality of life.

Table 1: Schematic view of study schedule with timing of study visits and content of each visit

**Visit**	**Screening**																
**Month**	**0**	**1**	**2**	**3**	**4**	**5**	**6**	**7**	**8**	**9**	**10**	**11**	**12**	**15**	**18**	**21**	**24**
Eligibility	X																
Alcohol screen	X			X			X			X			X	X	X	X	X
Karnofsky Performance status	X	X	X	X	X	X	X	X	X	X	X	X	X	X	X	X	X
Demographics	X																
Pregnancy test	X																
Medical history	X																
Informed consent	X																
EQ-5D	X			X			X			X			X	X	X	X	X
Adverse events/toxicity		X	X	X	X	X	X	X	X	X	X	X	X	X	X	X	X
Compliance (disulfiram)		X	X	X	X	X	X	X	X	X	X	X	X	X	X	X	X
Discontinuation		X	X	X	X	X	X	X	X	X	X	X	X	X	X	X	X
Considered drug interactions	X			X			X			X							
MRI Scan (incl RANO)	X			X			X			X			X	X	X	X	X
**Blood samples:**																	
Hematological and liver monitoring	X	X	X	X	X	X	X	X	X	X	X	X	X	X	X	X	X

- Alcohol screen relevant if treated with disulfiram
- QoL assessed with EQ-5D until progression OR as long as on disulfiram treatment in case of treatment also beyond progression (i.e. together with 2nd line rescue therapy).
- Compliance assessed by disulfiram and copper tablet intake (by tablet count at controls) and patient self-reporting
- Routine assessment of adverse events indicated here as long as on active treatment either with alkylating chemotherapy (control group) or disulfiram (intervention group)
- Blood samples as indicated here with hematological and liver monitoring relevant as long as on active treatment (Hemoglobin, thrombocytes, leukocytes with differential count, ASAT, ALAT, GT, ALP, bilirubin, INR). The interval for patients treated with lomustine/PCV is 6 weeks, while 4 weeks is clinical routine in the case of temozolomide

5. Safety

5.1. General remark on safety of interventional treatment

In the drug information on disulfiram (www.felleskatalogen.no and www.fass.se) it is indicated that severe organic brain disease is a contraindication. This may be true in the treatment of alcohol dependency, but studies on compassionate use in glioblastoma patients describes this as well tolerated.[18] Consequently, there is reason to believe that in the clinical setting without alcohol dependency this is not a significant clinical problem.

Concerning the nutritional supplement with copper this is generally not considered a problem in the dose described here (2,5 mg).[21] Nevertheless, potential adverse reaction of copper intoxication includes salivation, epigastric pain, nausea, vomiting, diarrhea, renal and hepatic injury.

Routine blood samples in all patients every 4-6 weeks (depending on lomustine or temozolomide based treatment; see above) to screen hematological and liver function. This is

considered clinical routine.

In addition, due to the well-known profile of disulfiram we will specifically look for

1. Uncontrolled seizures
2. Psychosis or delirium not induced by increasing corticosteroids

5.2. Definition adverse event

An adverse event is any untoward medical occurrence in a subject to whom a medicinal product has been administered, including occurrences which are not necessarily caused by or related to that product. The procedure of adverse event reporting is covered in detail by attachment 8.

Due to expected toxicity of the treatments and symptoms related to cancer diagnosis, *only grade 3, 4 and 5 (CTCAE) or grade ≥ 2 for infections* should be reported.

5.3. Toxicity

Adverse events and toxicities must be graded according to the NCI Common Toxicity Criteria for adverse events (NCI-CTCAE) Version 4.0 (http://ctep.cancer.gov/reporting/ctc.html).

5.4. A Serious Adverse Event (SAE)

A medical occurrence or effect that at any dose falls in one or more of the following categories:

- Results in death
- Is life-threatening
- Requires hospitalization or prolongation of existing inpatients' hospitalization. Hospitalization refers to a situation whereby an AE is associated with unplanned overnight admission into hospital.
- Results in persistent or significant disability or incapacity
- Is a medically significant adverse event

It is the responsibility of the local investigator to collect all the above-mentioned adverse events. Adverse events unequivocally due to standard of care treatment (see attachment 8) will not be reported as adverse events. Also, for definitions of categories of SAEs, also see attachment 8.

Still, events based on the knowledge of the disease in question and expected clinical course some events otherwise classified as serious are not considered/classified as SAE in this study. The following is a list of SAEs, which do not require to be reported as SAEs:

- Events based on the knowledge of the disease in question and expected clinical course (table 2).
- If a patient is admitted to hospital with a documented cancer related problem then this will not be reported as a SAE (table 2).

Table 2: Serious adverse events not to be reported

**Events**	**Description**

Disease Related Events	• Hospitalization or death due to disease progression<br>• Hospitalization for planned investigations<br>• Blood/platelet transfusion/fluid transfusion
Other	• Hospitalization for study drug administration, palliative care, terminal care or elective surgery

In every reportable case of SAE the investigator is responsible for ensuring that reporting to the Sponsor occurs no later than 24 hours after any of the site staff become aware of the event. Initial reports should be followed as soon as possible by detailed written reports describing the nature of the event and clinical consequences.

5.5. Serious Adverse Reaction (SAR) and Suspected Unexpected Serious Adverse Reactions (SUSAR)

A SAR is an SAE that may be related to trial treatment. The assessment of "relatedness" is primarily the responsibility of the Principal Investigator at site or agreed designee (table 3).

SUSAR are reactions where disulfiram (or copper supplement) may be suspected as the reason for an unexpected SAR. Both SAR and SUSAR are considered more serious and have to be reported to the sponsor immediately (within 24 hours). The sponsor is responsible to report to the respective national authorities within their time limits.

SAEs that will be considered related (i.e. SAR or SUSAR) include any SAE that is documented as possibly, probably or definitely related to protocol treatment.

Table 3: The assessment of relatedness is made using the following:

**Description**
There is no evidence of any causal relationship.
There is some evidence to suggest a causal relationship (e, g. the event occurs within a reasonable time after administration of the trial medication). However the influence of other factors may have contributed to the event (e.g. the patient's clinical condition, other concomitant treatments).
There is evidence to suggest a causal relationship and the influence of other factors is unlikely.
There is clear evidence to suggest a causal relationship and other possible contributing factors can be ruled out.

SAR and SUSAR are considered more serious because of the relatedness and have to be reported to the sponsor immediately without delay (within 24 hours). The sponsor is responsible to report to the respective national authorities within their time limits.
For an unexpected and serious adverse event that was lethal or life threatening, immediate and no later than 7 days after being notified the sponsor has to report to authorities in all involved EEA countries. Relevant follow-up information concerning further clinical progress has to be given no later than 8 days after the initial report.
Also, sponsor is obliged to send immediately and no later than 15 days after being notified of an unexpected and serious adverse event that is not lethal or life threatening to authorities in all involved EEA countries.
In addition, sponsor is obliged to report back to local investigators of suspected adverse

events that are serious and unexpected (i.e. SUSAR).

5.6 Special considerations for women of childbearing potential

As mentioned in exclusion criteria 6 and 7 pregnant patients or patients that are breastfeeding cannot be included in this trial. As stated, women of childbearing potential must also provide a negative pregnancy test before enrollment. Further, women of childbearing potential have to use a highly effective birth control method as defined by the European Heads of Medicines Agencies, Clinical Trial Facilitation Group document concerning "Recommendations related to contraception and pregnancy testing in clinical trials", paragraph 4.1, page 9 (http://www.hma.eu/fileadmin/dateien/Human_Medicines/01-About_HMA/Working_Groups/CTFG/2014_09_HMA_CTFG_Contraception.pdf).

6. Trial conduct

This study will be conducted in compliance with the protocol and according to Good Clinical Practice guidelines and applicable regulatory standards. Specifically, the Swedish and Norwegian ethical committees and the national medicine agencies need to approve the study. Further, the study will be conducted in accordance with the latest Declaration of Helsinki.[26]

7. Quality assurance and control

7.1. Case report forms

All data will be entered in the eCRF at the local study site. The Unit for Applied Clinical Research at the Norwegian University for Science and Technology (Trondheim, Norway) will manage the eCRF.

7.2. Patient security

All patient information is handled using patient ID that is not logically connected to any personal data. It is possible to "decode" back to personal data using a name list stored securely at the respective study sites. In the eCRF patients will be identified with a study participation number.

7.3. Specification of source documents

The following documents are source documents

- Patient hospital records
- Radiology reports
- MRI scans
- Lab reports
- Chemotherapy registers
- Radiotherapy registers
- The quality of life questionnaires

7.4. Archiving

All data will be stored at the participating sites for 15 years. In addition the electronic data will be kept during this time.

7.5. Data management

The CRFs in this trial are implemented electronically (i.e. eCRF) using a dedicated software developed at the Unit for Applied Clinical Research at the Norwegian University for Science and Technology (NTNU). This solution is at present (December 2015) used by more than 120 ongoing studies.

All data entered into the eCRF are stored at two separate servers at the NTNU. These servers are protected by the general firewall at the university and then by another firewall dedicated to protect clinical research data. The server that stores the data is not the same server as the one in contact with the Internet. There are several further safety measures not to be exposed. There are backups taken every 24 hour throughout the years. The responsibility for this system lies with the Unit for Applied Clinical Research at NTNU, Trondheim, Norway.

7.6. Analysis

At time of analysis, data files will be extracted from the database into statistical packages to be analyzed.

7.7. Monitoring

All source data must be accessible for auditing and monitoring. Monitor will maintain patient confidentiality.

Norway:
Unit for Applied Clinical Research at the Norwegian University for Science and Technology (NTNU). Phone: (+47) 72 57 11 09. Postal address: AKF, Det medisinske fakultet, Institutt for Kreftforskning og molekylær medisin, Postboks 8905, N-7491 Trondheim

Sweden:
Klinisk prövningsenhet, Verksamhet onkologi, Blå Stråket 2, Sahlgrenska Universitetssjukhuset, 413 45 Göteborg, Sweden, Phone: +46 (0)31-342 7654

For details, see attachment 9.

7.8. Inspection

Authorities have the right to perform inspections as well as the monitor. The inspector will have access to all relevant source data and study material. The local responsible investigator will answer any questions arising. However, the sponsor/principal investigator will be available for questions/dialogue in the immediate period following such inspections as well. All involved parties will keep the patient data strictly confidential.

7.9. Premature Termination of the Study

The sponsor may terminate the study prematurely for any reasonable cause. The Ethics committees and competent authorities will then be informed promptly. Conditions that may warrant termination include, but are not limited to:

- The discovery of an unexpected, significant, or unacceptable risk to the patients enrolled in the study.
- If competent authorities obtain information that raises doubts about the safety or scientific validity of the study, the competent authorities can suspend or prohibit the study.

8. Insurance
Patients will be insured according to national requirements.

Norway: www.laf.no (Legemiddelansvarsforeningen, LAF = the Drug Liability Association), see attachment 10. The sponsor has made a specific insurance that covers all Norwegian study participants.

Sweden: The patients are covered by “läkemedelsförsäkringen” (www.lakemedelsforsakringen.se) and “patientforsäkringen” (www.lof.se). For details, see attachment 10.

9. Regulatory requirements
The respective ethical committees at the study sites need to approve the study. In addition, the medical product agencies (the Swedish Medical Products Agency (MPA) and the Norwegian Medicines Agency (NMA)), have to authorize the study. Enrolment of patients will not start until approval has been received from both the Ethics Committee(s) and Competent authorities.

It may be desirable to make changes in the study at a later stage. In that case, the medical product agencies and ethical committees must approve significant changes in the protocol before they are implemented. We will use the form that is common across the EU (Substantial Amendment Notification Form) in reporting to the medical product agencies. A substantial change, is for example, change of the main purpose, primary or secondary variables, method to measure the primary endpoint, change of investigational medicinal products or dosage.

10. Cost
Due to the cheap treatment and the small change in follow-up routines the costs will be taken through regular clinical practice or from research grants provided by sponsor.

Administration of project, data monitoring, randomization process, data management, statistics and radiology services will be covered by sponsor through research grants. The project has a total budget of approximately 4 Mill SEK.

11. Estimated progress

	Year	Time of year
Ethical approvals (Norway/Sweden)	2016	Spring
Swedish Medical Products Agency (MPA)	2016	Spring
Norwegian Medicines Agency (NMA)	2016	Spring
Trial registration (clinicaltrials/EudraCT)	2016	Spring
Insurance	2016	Spring
Financing in place to start	2016	Autumn
Patient enrollment start	2017	Winter
Patient enrollment finish	2021	Autumn
6-month patient follow-up finish	2022	Spring
Interpretation, drafting, revision	2022	Autumn

Publication	2023	Spring
2 year follow up finish	2021	Winter
Follow-up publication	2021	Autumn

12. Contact information

12.1. Sponsor/principal investigator:

Department of Neurosurgery, Sahlgrenska University Hospital, Gothenburg where principal investigator Asgeir S. Jakola has the responsibility of this research project.

Asgeir S. Jakola has the following affiliations:

1. Department of Neurosurgery, Sahlgrenska University Hospital, Gothenburg, Blå Stråket 5, vån 3, 41345 Göteborg, Sweden
2. Department of Neurosurgery, St.Olavs Hospital, Trondheim, (Olav Kyrres Gate, 7006 Trondheim), Norway

Phone: 0047-91805300 and 0046-725559101
E-mail: legepost@gmail.com and Asgeir.jakola@vgregion.se

12.2. Monitor

Norway:
Unit for Applied Clinical Research at the Norwegian University for Science and Technology (NTNU). Phone: (+47) 72 57 11 09. Postal address: AKF, Det medisinske fakultet, Institutt for Kreftforskning og molekylær medisin, Postboks 8905, N-7491 Trondheim

Sweden:
Klinisk prövningsenhet, Verksamhet onkologi, Blå Stråket 2, Sahlgrenska Universitetssjukhuset, 413 45 Göteborg, Sweden, Phone: +46 (0)31-342 7654

12.3. On-site responsible physicians/investigators

The on-site responsible researchers and their responsibilities are outlined in attachment 11

13. References

1. Schwartzbaum JA, Fisher JL, Aldape KD, Wrensch M. Epidemiology and molecular pathology of glioma. *Nat Clin Pract Neurol.* 2006;2(9):494-503; quiz 491 p following 516.
2. Ronning PA, Helseth E, Meling TR, Johannesen TB. A population-based study on the effect of temozolomide in the treatment of glioblastoma multiforme. *Neuro Oncol.* 2012;14(9):1178-1184.

3. Stupp R, Mason WP, van den Bent MJ, et al. Radiotherapy plus concomitant and adjuvant temozolomide for glioblastoma. *N Engl J Med.* 2005;352(10):987-996.
4. Hegi ME, Diserens AC, Gorlia T, et al. MGMT gene silencing and benefit from temozolomide in glioblastoma. *N Engl J Med.* 2005;352(10):997-1003.
5. Taal W, Oosterkamp HM, Walenkamp AM, et al. Single-agent bevacizumab or lomustine versus a combination of bevacizumab plus lomustine in patients with recurrent glioblastoma (BELOB trial): a randomised controlled phase 2 trial. *Lancet Oncol.* 2014;15(9):943-953.
6. Collins FS. Mining for therapeutic gold. *Nature reviews. Drug discovery.* 2011;10(6):397.
7. Chick J. Safety issues concerning the use of disulfiram in treating alcohol dependence. *Drug safety.* 1999;20(5):427-435.
8. Borup C, Kaiser A, Jensen E. Long-term Antabuse treatment: tolerance and reasons for withdrawal. *Acta psychiatrica Scandinavica. Supplementum.* 1992;369:47-49.
9. Dufour P, Lang JM, Giron C, et al. Sodium dithiocarb as adjuvant immunotherapy for high risk breast cancer: a randomized study. *Biotherapy (Dordrecht, Netherlands).* 1993;6(1):9-12.
10. Nechushtan H, Hamamreh Y, Nidal S, et al. A phase IIb trial assessing the addition of disulfiram to chemotherapy for the treatment of metastatic non-small cell lung cancer. *The oncologist.* 2015;20(4):366-367.
11. Chen D, Cui QC, Yang H, Dou QP. Disulfiram, a clinically used anti-alcoholism drug and copper-binding agent, induces apoptotic cell death in breast cancer cultures and xenografts via inhibition of the proteasome activity. *Cancer Res.* 2006;66(21):10425-10433.
12. Kast RE, Boockvar JA, Bruning A, et al. A conceptually new treatment approach for relapsed glioblastoma: coordinated undermining of survival paths with nine repurposed drugs (CUSP9) by the International Initiative for Accelerated Improvement of Glioblastoma Care. *Oncotarget.* 2013;4(4):502-530.
13. Liu P, Brown S, Goktug T, et al. Cytotoxic effect of disulfiram/copper on human glioblastoma cell lines and ALDH-positive cancer-stem-like cells. *Br J Cancer.* 2012;107(9):1488-1497.
14. Loo TW, Bartlett MC, Clarke DM. Disulfiram metabolites permanently inactivate the human multidrug resistance P-glycoprotein. *Molecular pharmaceutics.* 2004;1(6):426-433.
15. Rae C, Tesson M, Babich JW, Boyd M, Sorensen A, Mairs RJ. The role of copper in disulfiram-induced toxicity and radiosensitization of cancer cells. *J Nucl Med.* 2013;54(6):953-960.
16. Triscott J, Lee C, Hu K, et al. Disulfiram, a drug widely used to control alcoholism, suppresses the self-renewal of glioblastoma and over-rides resistance to temozolomide. *Oncotarget.* 2012;3(10):1112-1123.
17. Westhoff MA, Zhou S, Nonnenmacher L, et al. Inhibition of NF-kappaB signaling ablates the invasive phenotype of glioblastoma. *Molecular cancer research : MCR.* 2013;11(12):1611-1623.
18. Kast RE, Karpel-Massler G, Halatsch ME. CUSP9* treatment protocol for recurrent glioblastoma: aprepitant, artesunate, auranofin, captopril, celecoxib, disulfiram, itraconazole, ritonavir, sertraline augmenting continuous low dose temozolomide. *Oncotarget.* 2014;5(18):8052-8082.

19. Paranjpe A, Zhang R, Ali-Osman F, Bobustuc GC, Srivenugopal KS. Disulfiram is a direct and potent inhibitor of human O6-methylguanine-DNA methyltransferase (MGMT) in brain tumor cells and mouse brain and markedly increases the alkylating DNA damage. *Carcinogenesis.* 2014;35(3):692-702.
20. Huang J, Campian JL, Gujar AD, et al. A phase I study to repurpose disulfiram in combination with temozolomide to treat newly diagnosed glioblastoma after chemoradiotherapy. *J Neurooncol.* 2016.
21. European Food Safety Authority; Tolerable upper intake levels for vitamins and minerals 2006; http://www.efsa.europa.eu/sites/default/files/efsa_rep/blobserver_assets/ndatolerableuil.pdf.
22. Batchelor TT, Mulholland P, Neyns B, et al. Phase III randomized trial comparing the efficacy of cediranib as monotherapy, and in combination with lomustine, versus lomustine alone in patients with recurrent glioblastoma. *J Clin Oncol.* 2013;31(26):3212-3218.
23. The. EuroQol Group: (1990) EuroQol - a new facility for the measurement of health-related quality of life. *Health Policy.*16(3):199-208.
24. Stensjoen AL, Solheim O, Kvistad KA, Haberg AK, Salvesen O, Berntsen EM. Growth dynamics of untreated glioblastomas in vivo. *Neuro Oncol.* 2015.
25. Khan I, Sarker SJ, Hackshaw A. Smaller sample sizes for phase II trials based on exact tests with actual error rates by trading-off their nominal levels of significance and power. *Br J Cancer.* 2012;107(11):1801-1809.
26. World Medical Association Declaration of Helsinki: ethical principles for medical research involving human subjects. *Jama.* 2013;310(20):2191-2194.

14. Signature pages

Sponsor and co-ordinating investigator

Asgeir S. Jakola, MD, PhD
I have read and understood this trial protocol and will work according to this protocol.

____

Date and signature

On-site responsible physicians/investigators

Principal investigator Göteborg:
Katja Werlenius, MD, oncologist.

I have read and understood this trial protocol and agree to conduct the trial as set out in this protocol, the current version of the World Medical Association Declaration of Helsinki, GCP guidelines and locally legally applicable requirements

____

Date and signature

Principal investigator Linköping:
Munila Mudaisi, MD, oncologist.

I have read and understood this trial protocol and agree to conduct the trial as set out in this protocol, the current version of the World Medical Association Declaration of Helsinki, GCP guidelines and locally legally applicable requirements

____

Date and signature

Principal investigator Stockholm:
Sofia Hylin, MD, neurologist.

I have read and understood this trial protocol and agree to conduct the trial as set out in this protocol, the current version of the World Medical Association Declaration of Helsinki, GCP guidelines and locally legally applicable requirements

____

Date and signature

Principal investigator Lund:
Sara Kinhult, MD, PhD, oncologist.

I have read and understood this trial protocol and agree to conduct the trial as set out in this protocol, the current version of the World Medical Association Declaration of Helsinki, GCP guidelines and locally legally applicable requirements

____

Date and signature

Principal investigator Uppsala:
Magnus Lindskog, MD, PhD, oncologist.

I have read and understood this trial protocol and agree to conduct the trial as set out in this protocol, the current version of the World Medical Association Declaration of Helsinki, GCP guidelines and locally legally applicable requirements

____

Date and signature

Principal investigator Jönköping:
Michael Strandéus, MD, oncologist.

I have read and understood this trial protocol and agree to conduct the trial as set out in this protocol, the current version of the World Medical Association Declaration of Helsinki, GCP guidelines and locally legally applicable requirements

____

Date and signature

Principal investigator Trondheim:
Tora S. Solheim, MD, PhD, oncologist.

I have read and understood this trial protocol and agree to conduct the trial as set out in this protocol, the current version of the World Medical Association Declaration of Helsinki, GCP guidelines and locally legally applicable requirements

____

Date and signature

Principal investigator Örebro:
David Löfgren, MD, oncologist.

I have read and understood this trial protocol and agree to conduct the trial as set out in this protocol, the current version of the World Medical Association Declaration of Helsinki, GCP guidelines and locally legally applicable requirements

____

Date and signature

Principal investigator Oslo (Radiumhospitalet):
Henriette Magelssen, MD, PhD, oncologist.

I have read and understood this trial protocol and agree to conduct the trial as set out in this protocol, the current version of the World Medical Association Declaration of Helsinki, GCP guidelines and locally legally applicable requirements

____

Date and signature

15. Attachments

Attachment 1: Quality of life. EuroQol 5D
Attachment 2: Common Terminology Criteria for Adverse Events (CTCAE) version 4.0
Attachment 3: Evaluation of end-points
Attachment 4: Clarification on progression on MRI
Attachment 5: Karnofsky Performance Status
Attachment 6a and 6b: Informed consent in Swedish and Norwegian
Attachment 7: Interim analysis and monitoring committee
Attachment 8: Adverse event reporting
Attachment 9: Details on study monitoring
Attachment 10: Details on insurance
Attachment 11: Responsibilities of key participants

Statistical Analysis Plan

Final

Protocol version 2.1, dated 18 feb 2019

DIRECT Glioblastoma

DIRECT (DIsulfiram REsponse as add-on to ChemoTherapy in recurrent) Glioblastoma: A randomized controlled trial

2020-06-01

Author

Name/Title:	
Nils-Gunnar Pehrsson / Senior Biostatistician, Statistiska Konsultgruppen	
Signature: Nils-G. Pehrsson	Date: 2020-06-01

Approvals

Name/Title:	
Asgeir S Jakola / Sponsor, Project leader	
Signature: [signature]	Date: 2020-06-09
Name/Title:	
Katja Werlenius / PI Göteborg	
Signature: [signature]	Date: 2020-06-17

Revisions

Version	Description of Changes	Date
x.x		yyyy-mm-dd

Table of Contents

1 Study Details 5
1.1 Study Objectives 5
1.2 Study Design 6
1.3 Treatment Groups 6
1.4 Sample Size 7
2 Study Populations 7
2.1 Definition of Study Populations 7
2.1.1 Intent-to-Treat Population (Full Analysis Set) 7
2.1.2 Per-Protocol Population 7
2.1.3 Safety Population 7
3 Study Variables 7
3.1 Baseline Variables 7
3.1.1 Demographics and Baseline Characteristics 7
3.2 Efficacy Variables 8
3.2.1 Primary Efficacy Variable 8
3.2.2 Secondary Efficacy Variables Secondary efficacy variables will be: 8
3.3 Safety Variables 8
3.3.1 Exposure of Study Drug 8
3.3.2 Adverse Events 8
• Reportable AE 8
3.3.3 Physical Examination 9
4 Statistical Methodology 9
4.1 General Methodology 9
4.2 Patient Disposition and Data Sets Analyzed 10
4.3 Protocol Violations/Deviations 10
4.4 Demographics and Baseline Characteristics 10
4.5 Efficacy Analyses 10
4.5.1 Primary Efficacy Analysis 10
4.5.2 Secondary Efficacy Analyses 10
4.6 Safety Analyses 11
4.6.1 Exposure of Study Drug 11
4.6.2 Adverse Events 11
4.7 Exploratory risk factor analyses 11
4.8 Exploratory interaction analyses, to find promising subgroups 11

5 Interim Analyses ... 12
6 Changes of Analysis from Protocol ... 12
7 Listing of Table, Figures and Listings ... 12
7.1 Listing of Tables ... 12
7.2 Listing of Figures ... 13
7.3 Listing of Listings ... 13

1 STUDY DETAILS

1.1 Study Objectives

Disulfiram (Antabus®) is a well-tolerated, cheap, generic drug that has been in use since the 1950s to treat alcoholism. There is now an increasing amount of independent preclinical data to support disulfiram as an anticancer agent. In glioblastomas add-on treatment to alkylating agents may offer additional benefit due to O^6-methylguanine methyltransferase (MGMT, a DNA-repair enzyme) inhibition. The potency of disulfiram as an anticancer agent seems strengthened by copper. There is now anecdotal clinical evidence of disulfiram as an anticancer, for instance with improved survival in lung cancer patients. Also, disulfiram was positively associated with survival in a registry study. Collectively these results indicate a clinical meaningful response.

Our objective is to investigate disulfiram and copper-supplement as add-on treatment in patients with glioblastoma undergoing treatment at time of recurrence with alkylating chemotherapy.

ClinicalTrials.gov Identifier: NCT02678975

The primary objective for this study is survival at 6 months

The secondary objectives for this study are the following:

- Actual survival analyzed at 9, 12 and 24 months.
- Median progression free survival
- 6 and 12 month progression free survival
- Median overall survival
- Health-related quality of life
- Volumetric expansion
- Toxicity/safety

1.2 Study Design

Multicenter, open labeled randomized controlled trial with parallel group design (1:1). With survival as end-point, but a more liberal power calculation, this is a phase II/III trial.

**Visit**	**Screening**																
**Month**	**0**	**1**	**2**	**3**	**4**	**5**	**6**	**7**	**8**	**9**	**10**	**11**	**12**	**15**	**18**	**21**	**24**
Eligibility	X																
Alcohol screen	X			X			X			X			X	X	X	X	X
Karnofsky Performance status	X	X	X	X	X	X	X	X	X	X	X	X	X	X	X	X	X
Demographics	X																
Pregnancy test	X																
Medical history	X																
Informed consent	X																
EQ-5D	X			X			X			X			X	X	X	X	X
Adverse events/toxicity		X	X	X	X	X	X	X	X	X	X	X	X	X	X	X	X
Compliance (disulfiram)		X	X	X	X	X	X	X	X	X	X	X	X	X	X	X	X
Discontinuation		X	X	X	X	X	X	X	X	X	X	X	X	X	X	X	X
Considered drug interactions	X			X			X			X							
MRI Scan (incl RANO)	X			X			X			X			X	X	X	X	X
**Blood samples:**																	
Hematological and liver monitoring	X	X	X	X	X	X	X	X	X	X	X	X	X	X	X	X	X

1.3 Treatment Groups

The active group with Disulfiram 400 mg once daily in addition to copper supplement (2 mg once daily) and alkylating chemotherapy Will be named Disulfiram+ CU+Chemo
The control group with Alkylating chemotherapy will be named Chemo in the TFLs

1.4 Sample Size

Based on the assumption that the treatment group will have an improvement in proportion achieving 6-month survival from 60 % to 80 % we need a final sample size of 128 in total (64 in each group; alpha 0.1, power 80 % and two sided test, see below). Considering 10 % attrition the actual included sample need to be **142 with 71 patients included in each group.**

Sample size (without continuity correction)

	N	Alive	Dead
disulfiram	64	51	13
controls	64	38	26
TOTAL	128	89	39

http://www.sample-size.net/sample-size-proportions/

alpha 0.1, two sided test, RR: 1.3333, power 80 %

60 % chance of survival versus 80 % chance of survival at 6 months

2 STUDY POPULATIONS

2.1 Definition of Study Populations

2.1.1 Intent-to-Treat Population (Full Analysis Set)

All randomized subjects will be included in the Intent-to-Treat (ITT) population.

2.1.2 Per-Protocol Population

All randomized subjects with no major protocol violations will be included in the Per Protocol (PP) population. The final decisions regarding the PP population will be taken at the Clean File meeting before the database lock.

2.1.3 Safety Population

All enrolled subjects who received at least one dose of randomised investigational product (IP) will be included in the safety population.

3 STUDY VARIABLES

3.1 Baseline Variables

3.1.1 Demographics and Baseline Characteristics

Age, sex, Karnofsky performance status, surgical procedure at initial treatment (biopsy vs resection), MGMT status (positive, negative, unknown), radiotherapy at initial treatment

(hypofractioned, normofractioned, none), chemotherapy at initial treatment (concomitant and adjuvant, other, none), type of chemotherapy (TMZ vs other), main tumor location, type of chemotherapy now indicated (TMZ, CCNU, PCV, Carmustine, other), radiotherapy for recurrence, surgery for recurrence, EQ-5D 3L index and VAS at baseline,

3.2 Efficacy Variables

3.2.1 Primary Efficacy Variable

Primary efficacy variable will be survival (Yes/No) at 6 months follow-up from randomization

3.2.2 Secondary Efficacy Variables

Secondary efficacy variables will be:

- Survival (Yes/No) at 9, 12 and 24 months follow-up from randomization
- Progression free survival: Time from randomization to progression or death
- Death or progression(Yes/No) at 6 and 12 months follow-up from randomization
- Survival: Time from randomisation to death
- Health-related quality of life: EQ-5D, Change from baseline continuous variable
- Volumetric expansion: as % daily change in tumor volume (volume baseline and volume first scan) Change from baaseline

3.3 Safety Variables

3.3.1 Exposure of Study Drug

Duration of therapy will be measured for each treatment.
Compliance of investigational treatment will be measured for each visit.

3.3.2 Adverse Events

- *Reportable AE*
- *SAE*

3.3.3 *Physical Examination*

Specify all variables and timepoints when to be analyzed and any algorithms needed for calculation of the variables.

If applicable, specify how baseline value is to be defined.

4 STATISTICAL METHODOLOGY

4.1 General Methodology

All the main analyses will be performed on the ITT population and supported analyses will be performed on the PP-population. The primary efficacy variable and all other dichotomous variables will be analysed with Chi-square test between the two groups.
All unadjusted survival analyses between the two randomised groups will be performed with log-rank test. Hazard ratios (HR) with 95% confidence interval (CI) will be calculated with Cox proportional hazard regression models. Kaplan-Meier curves will describe the survival function and estimate medians and percentiles. In addition event rates will be given.
All included subjects will be followed in the survival analyses at least two years, but most of the subjects will be followed for longer time. The censored time for all subjects survived will be the date when the last patient have been followed for two years. For all other types of efficacy variables the subjects will be followed up to two year visit.

For comparison between two groups Fisher's non-parametric permutation test will be used for continuous variables and Mantel-Haenszel Chi-square test for ordered categorical variables.

Mean difference between the two randomised groups with 95% CI will be the main results for dichotomous and continuous variables. For dichotomous outcome variables relative risk (RR) with 95% CI will also be calculated.
No imputation of data will be performed.

If baseline predictors for progression and death differ clinically and statistically between the two randomised groups complementary analyses will be performed adjusted for these baseline confounders. The proportional hazard assumption will be investigated for variables included in the Cox proportional hazard model. If some of the confounders do not fulfil the proportional assumption, they will be stratified for in the Cox proportional hazard model.

Adjustment for confounders will be performed with Cox proportional hazard model for survival analyses, with multiple logistic regression for dichotomous variables and with analysis of covariance for continuous variables.

Distributions of continuous variables will be given as mean, SD, median, min and max. For categorical variables number and percentages will be given.

The distribution of continuous variables measured over time will be given for each visit by randomisation group both in tables and figures. Changes from baseline will also be given for these variables by group in both tables and figures.
For comparisons within each randomised group over time Fisher's pared non-parametric permutation test will be used for continuous variables and Sign test for dichotomous and ordered categorical variables.

In order to find risk factors for progression and death univariable and stepwise exploratory models will be investigated.

In order to find subgroups where Disulfiram and copper had good effect interaction analyses will be performed.

All tests will be two-tailed. Primary analyses will be conducted at 10% significance level and all other tests at 0.05 significance level. All analyses will be performed by using SAS® v9.2 (Cary, NC).

4.2 Patient Disposition and Data Sets Analyzed

The number of subjects included in each of the ITT, PP and safety populations will be summarized for each treatment group and overall. The number and percentage of subjects randomized and treated will be presented. Subjects who completed the study and subjects who withdrew from study prematurely will also be presented with a breakdown of the reasons for withdrawal by treatment group for the ITT, PP and safety populations.

4.3 Protocol Violations/Deviations

Major protocol deviations are those that are considered to have an effect on the analysis. A list of potential major protocol deviations will be generated programmatically from the data captured before the clean file meeting. The clinical monitors of the study will review the list and the finalisation of the major protocol deviations will be done at the clean file meeting.

The number of patients with major protocol deviations will be summarized per treatment group.

4.4 Demographics and Baseline Characteristics

Demographics and baseline characteristics will be summarized by treatment group for the ITT and PP populations and analyzed according to the methods described in section "General Methodology" above.

4.5 Efficacy Analyses

4.5.1 Primary Efficacy Analysis

Primary efficacy analyses will be the analysis of survival at six months between the Disulfiram+ CU+Chemo group compared the Chemo group with two-sided Chi-square test at significance level 0.10 on the ITT population. Mean percent differences with 95% CI together with relative risk (RR) with 95% CI. The actual percentages with 95% exact CI will also be given.

A sensitivity analyses will be performed with imputed data on survival at 6 months if lost to follow up. A complementary analysis of the primary analysis will be performed on the PP-population.

4.5.2 Secondary Efficacy Analyses

The secondary efficacy analyses will be the analyses of the secondary variables given in section 3.2.2 according to the statistical methods given in section 4.1 "General methodology" above.

4.6 Safety Analyses

4.6.1 Exposure of Study Drug

Duration of therapy will be summarized for each treatment. Compliance of investigational treatment will be summarized for each visit.

The summaries will be provided for safety population.

4.6.2 Adverse Events

Only treatment-emergent AEs 3 or higher (except infections with grade 2 or higher) according to CTCAE v 4.0 will be included in the summaries for safety population.

A summary of subjects reporting at least one of the following AEs will be presented in an overview table:

- Any AE 3 or higher (except infections; grade 2 or higher) according to CTCAE v 4.0
- Any SAE
- Any SAE by presumed causality
- Any SAE causing Death

Summaries per SOC and PT presenting n (%) of AEs and n (%) of subjects with at least one AE will be provided for:

- All AEs (includes all serious and non-serious AEs)
- All AEs by maximum reported intensity
- All AEs by causality
- All SAEs
- All AEs leading to discontinuation

4.7 Exploratory risk factor analyses

Find independent risk factors for primary efficacy variable survival at 6 months and other selected secondary outcomes with univariable and stepwise multivariable methods.

For dichotomous outcome variables logistic regression will be used and for survival analyses Cox proportional hazard model will be used.

4.8 Exploratory interaction analyses, to find promising subgroups

Exploratory interaction analyses between the two treatment groups and baseline variables for primary efficacy variable and selected secondary variables. For baseline variables with an interaction p-value subgroup analyses will be performed for that variables regarding the present outcome variables between the two randomised groups.

5 INTERIM ANALYSES

An DSMB will be appointed to look at the efficacy, safety and futility (low conditional power) in the study.

A DSMB charter will be agreed on between the DSMB members and the steering committee.

If futility is less than 0.20 then DSMB should recommend the study to stop.

If interim analyses lead to study closure, the full analyses according to this SAP will be performed.

6 CHANGES OF ANALYSIS FROM PROTOCOL

The following analyses have been added before database lock:

- Risk factors analysis for 6 months survival
- Interaction analyses to find subgroups with good treatment effect.

Interim analyses included primarily safety measures, but due to Covid-19 situation with pause in recruitment we decided to include also efficacy measures and postpone analysis to 71 patients with six-month follow-up period instead of performing the analyses when patient #71 was included.

7 LISTING OF TABLE, FIGURES AND LISTINGS

7.1 Listing of Tables

Table Number	Table Title
14.1.1	Patient Disposition and Data Sets Analyzed (ITT Population)
14.1.2	Protocol Deviations Leading to Exclusion from PP Population (ITT Population)
14.1.3.1	Demographics and Baseline Characteristics (ITT Population)
14.1.3.2	Demographics and Baseline Characteristics (PP Population)
14.1.4	Medical History (ITT Population)
14.1.5	Surgical History (ITT Population)
14.1.6.1	Prior Medications (ITT population)
14.1.6.2	Concomitant Medications (ITT population)
14.2.1.1	Primary Efficacy Analysis (ITT Population)
14.2.1.2	Primary Efficacy Analysis (PP Population)
14.2.2.1	Secondary Efficacy Analyses (ITT Population)
14.2.2.2	Secondary Efficacy Analyses (PP Population)
14.2.3	Risk factors analysis of six months survival (ITT population)
14.2.4.1	Interaction analyses between baseline variables and treatment (ITT population)
14.2.4.2	Subgroup analyses based on the interaction analyses (ITT population)
14.3.1	Compliance (Safety Population)

14.3.2.1	Summary of Adverse Events (Safety Population)
14.3.2.2	Adverse Events (Safety Population)
14.3.2.3	Serious Adverse Events, by System Organ Class and Preferred Term (Safety Population)
14.3.4.1	Descriptive Statistics for Laboratory Variables:

7.2 Listing of Figures

Figure Number	Table Title
14.2.1.1	Plot of survival (yes/No) over the study by group (ITT-population)
14.2.1.2	Plot of survival (yes/No) over the study by group (PP-population)
14.2.2.1	Kaplan-Meier curves by randomized group (ITT-population)
14.2.2.1	Kaplan-Meier curves by randomized group (PP-population)
14.2.3.1	Continuous secondary efficacy variables over time by randomized group (ITT-population)
14.2.3.2	Continuous secondary efficacy variables over time by randomized group (PP-population)
14.2.4.1	Change in continuous secondary efficacy variables from baseline over time by randomized group (ITT-population)
14.2.4.2	Change in continuous secondary efficacy variables from baseline over time by randomized group (PP-population)
14.2.5.1-14.2.5.x	Kaplan-Meier curves by randomized group for studied subgroups (ITT-population)

7.3 Listing of Listings

Listing number	Listing Title
16.2.1	Discontinued Patients
16.2.2	Patients with Important Protocol Deviations
16.2.3	Patients Excluded from the Efficacy Analysis
16.2.4.1	Demographics and Baseline Characteristics
16.2.4.2	Medical History
16.2.4.3	Surgical History
16.2.4.4	Prior and Concomitant Medications
16.2.5	Compliance and Drug Exposure
16.2.6	Efficacy Variables
16.2.7	Adverse Events
16.2.8	Laboratory Data
16.2.9	Vital Signs Data
16.2.10	ECG Data
