# Supplementary material for: Effect of Disulfiram and Copper Plus Chemotherapy vs Chemotherapy Alone on Survival in Patients With Recurrent Glioblastoma: A Randomized Clinical Trial
Source: JAMA Netw Open. 2023 Mar 31;6(3):e234149. doi: 10.1001/jamanetworkopen.2023.4149 (PMC10066460; doi:10.1001/jamanetworkopen.2023.4149)
Supplement: Supplement 2. — eTable 1. Baseline Characteristics in the Per-Protocol Population eTable 2. Treatment Characteristics in the Per-Protocol Population eFigure. Overall Survival in the Per-Protocol Population eTable 3. Summary of Adverse Events in the Safety Population [file jamanetwopen-e234149-s002.pdf]

Supplemental Online Content

Werlenius K, Kinhult S, Solheim TS, et al. Effect of disulfiram and copper plus chemotherapy vs chemotherapy alone on survival in patients with recurrent glioblastoma: a randomized clinical trial. *JAMA Netw Open.* 2023;6(3):e234149. doi:10.1001/jamanetworkopen.2023.4149

eTable 1. Baseline Characteristics in the Per-Protocol Population

eTable 2. Treatment Characteristics in the Per-Protocol Population

eFigure. Overall Survival in the Per-Protocol Population

eTable 3. Summary of Adverse Events in the Safety Population

This supplemental material has been provided by the authors to give readers additional information about their work.

© 2023 Werlenius K et al. *JAMA Network Open.*

eTable 1: Baseline characteristics in the Per-protocol population

	Standard of care (n=40)	Standard of care + Disulfiram-Copper (n=41)	Total (N=81)
Age, mean (SD), years	54.8 (11.3)	56.3 (12)	55.6 (11.6)
Sex, n (%)			
Male	26 (65)	33 (80)	59 (73)
Female	14 (35)	8 (20)	22 (27)
Karnofsky performance status, n (%)			
60%	3 (8)	2 (5)	5 (6)
70-80%	11 (28)	15 (37)	26 (32)
90-100%	26 (65)	24 (59)	50 (62)
Initial surgery, n (%)			
Resection	35 (88)	38 (93)	73 (90)
Biopsy	5 (13)	3 (7)	8 (10)
Initial radiotherapy with concurrent temozolomide, n (%)			
Yes	37 (93)	39 (95)	76 (94)
No[a]	3 (8)	2 (5)	5 (6)
Tumor characteristics, n (%)			
IDH1[b] wildtype	28 (70)	28 (68)	56 (69)
IDH1[b] mutated	3 (8)	4 (10)	7 (9)
IDH1[b] unknown	9 (23)	9 (22)	18 (22)
MGMT[c] un-methylated	15 (38)	21 (51)	36 (44)
MGMT[c] methylated	13 (33)	11 (27)	24 (30)
MGMT[c] unknown	12 (30)	9 (22)	21 (26)
Use of steroids at baseline, n (%)			
Yes	19 (48)	20 (49)	39 (48)
No	21 (53)	20 (49)	41 (51)
Missing		1 (2)	1 (1)

Percentages may not add up to 100 due to rounding.

[a]Other initial treatment than concurrent radiochemotherapy, only one patient did not receive temozolomide as part of the initial treatment, [b]isocitrate dehydrogenase, [c]O^6-methylguanine-DNA methyltransferase

© 2023 Werlenius K et al. *JAMA Network Open.*

eTable 2: Treatment characteristics in the Per-protocol population

	**Standard of care (n=40)**	**Standard of care + Disulfiram-Copper (n=41)**	**p-value**
Chemotherapy, n (%)			0.34
Temozolomide	11 (28)	14 (34)	
Lomustine	27 (68)	22 (54)	
PCV[a]	2 (5)	5 (12)	
Duration of chemotherapy, median (IQR), days	94 (60-220), n=39	60 (33-98), n=35	0.007
Radiotherapy for recurrence, n (%)	1 (3)	3 (7)	0.32
Surgery for recurrence, n (%)	10 (25)	15 (37)	0.19
Actual n per cell is provided in cases of missing values. Percentages may not add up to 100 due to rounding.<br><br>[a]Procarbazine, lomustine and vincristine			

© 2023 Werlenius K et al. *JAMA Network Open.*

eFigure: Overall survival in the Per-protocol population

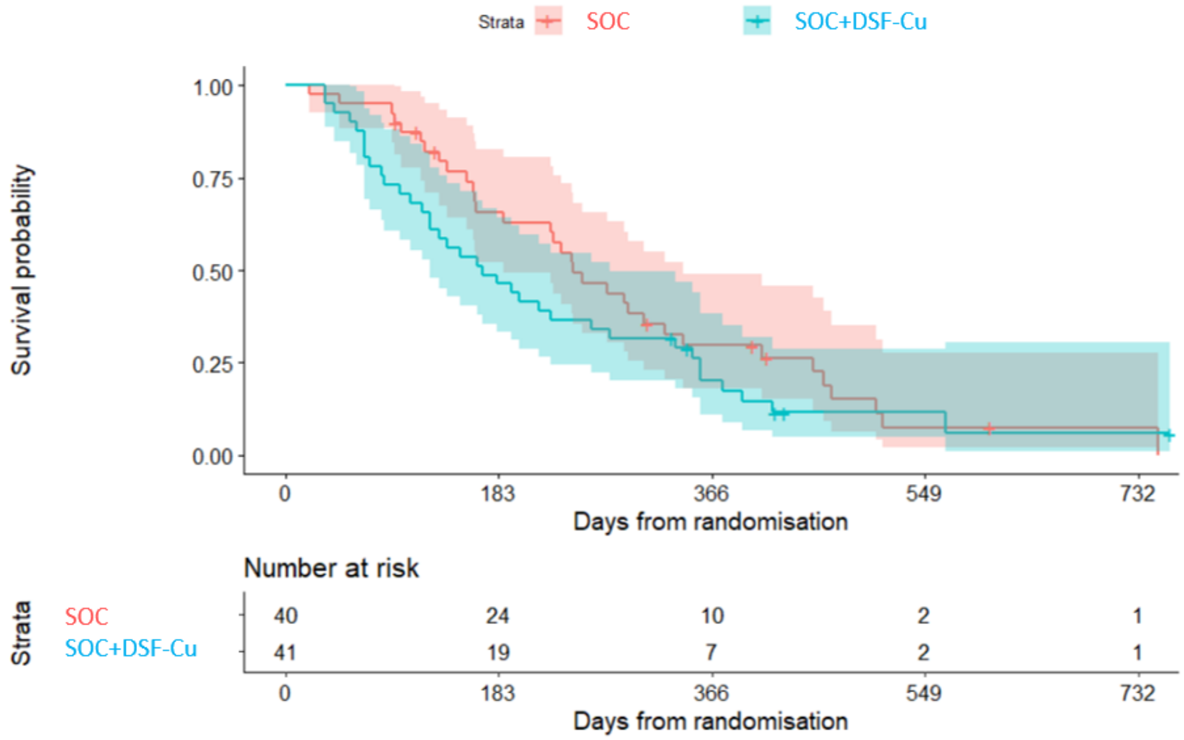

The difference between survival is not significant, p = 0.22, using a Cox proportional hazard method.

© 2023 Werlenius K et al. *JAMA Network Open.*

eTable 3. Summary of Adverse Events in the Safety Population

Safety population, summary of adverse events	Standard of care (n = 44)		Standard of care + disulfiram-copper (n = 41)		*P* value for patients with AE
	AEs, No.	Patients with AEs, No. (%)	AEs, No.	Patients with AEs, No. (%)	
AE CTCAE grade ≥3	17	5 (11)	31	14 (34)	.02
Any SAE	8	7 (16)	22	17 (41)	.02
SAEs related to Disulfiram-Copper	NA	NA	12	9 (22)	NA
SAEs leading to death	0	0	1	1 (2)	NA
AEs leading to discontinued Disulfiram-Copper treatment	NA	NA	11	10 (24)	NA

Abbreviations: AE, adverse event; CTCAE, Common Terminology Criteria for Adverse Events; NA, not applicable; SAE, serious adverse event.

© 2023 Werlenius K et al. *JAMA Network Open.*
