# Supplementary material for: Effect of Disulfiram and Copper Plus Chemotherapy vs Chemotherapy Alone on Survival in Patients With Recurrent Glioblastoma: A Randomized Clinical Trial
Source: JAMA Netw Open. 2023 Mar 31;6(3):e234149. doi: 10.1001/jamanetworkopen.2023.4149 (PMC10066460; doi:10.1001/jamanetworkopen.2023.4149)
Supplement: Supplement 3. — Data Sharing Statement [file jamanetwopen-e234149-s003.pdf]

Data Sharing Statement

Werlenius. Effect of Disulfiram and Copper Plus Chemotherapy vs Chemotherapy Alone on Survival in Patients With Recurrent Glioblastoma. *JAMA Netw Open*. Published March 31, 2023. doi:10.1001/jamanetworkopen.2023.4149

Data
Data available: No

Additional Information
Explanation for why data not available: Anonymous data can be provided upon reasonable request.
